# Direct RNA sequencing reveals m6A modifications and isoform changes in SARS-CoV-2-infected HEK cells

**DOI:** 10.1099/acmi.0.001019.v3

**Published:** 2025-09-17

**Authors:** Ilhan Cem Duru, Zlatka Plavec, Anne Ylinen, Pia Laine, Martyn James, Lotta Riihimäki, Sarah J. Butcher, Maria Anastasina, Petri Auvinen

**Affiliations:** 1 Institute of Biotechnology, Helsinki Institute of Life Sciences, University of Helsinki, Helsinki, Finland; 2 Faculty of Biological and Environmental Sciences, Molecular and Integrative Bioscience Research Programme, University of Helsinki, Helsinki, Finland

**Keywords:** COVID-19, direct RNA sequencing, RNA methylation, SARS-CoV-2

## Abstract

The severe acute respiratory syndrome coronavirus 2 (SARS-CoV-2) infection triggers complex host responses, including alterations in RNA transcription and modification. Understanding these changes is crucial for elucidating viral pathogenesis and identifying potential therapeutic targets. We used direct RNA sequencing to comprehensively profile the transcriptomic and epitranscriptomic landscapes of human HEK-AT cells infected with SARS-CoV-2 at 8 h post-infection, compared to mock controls. We analysed viral and host transcriptomes, focusing on gene and transcript expression, isoform usage and RNA m6A modifications. Viral RNA sequencing reads showed 3′ end-biassed coverage indicative of subgenomic RNA synthesis, with high expression of *N* gene subgenomic RNA reads. Sixteen m6A modification sites were consistently identified in the viral genome, primarily within the *ORF1ab* and *S* genes. In the human transcriptome, we found 254 positions with significantly altered m6A modification rates, with 119 showing decreased modification and 135 showing increased modification in infected cells. Genes with decreased m6A modifications were enriched in the neurotrophin signalling pathway. Transcript-level analysis identified 19 upregulated and 12 downregulated transcripts. Notably, transcript discovery and quantification revealed a novel isoform of the *HIST1H2BK* gene, which was significantly more expressed in infected cells compared to mock controls. Isoform switching analysis revealed 24 significant switches involving 21 genes, implicating mitochondrial reprogramming and immune-related pathways. In conclusion, this study provides a detailed, direct RNA sequencing-based characterization of host–virus RNA interactions, revealing key insights into SARS-CoV-2 infection mechanisms and potential therapeutic targets.

## Data Summary

All sequencing data have been deposited in the European Nucleotide Archive under accession code PRJEB85775. Individual sequence accession numbers are listed below:

**Table:** 

Sample	Raw FAST5	Fastq basecalled
Mock HEK 1	ERR14708101	ERR14375867
Mock HEK 2	ERR14710019	ERR14375868
Mock HEK 3	ERR14710020	ERR14375870
SARS-CoV-2 infected 8 h HEK 1	ERR14710021	ERR14375871
SARS-CoV-2 infected 8 h HEK 2	ERR14710022	ERR14375872
SARS-CoV-2 infected 8 h HEK 3	ERR14710023	ERR14375873

## Introduction

The severe acute respiratory syndrome coronavirus 2 (SARS-CoV-2) pandemic has highlighted the critical importance of understanding viral–host interactions at the molecular level. The rapid evolution of SARS-CoV-2 variants and their varying transmission rates presents ongoing challenges for diagnostic approaches and therapeutic interventions [1,2]. While significant progress has been made in characterizing SARS-CoV-2 protein functions and cellular entry mechanisms [3,4], our understanding of how the virus modifies host cell RNA biology remains incomplete.

RNA modifications, particularly N6-methyladenosine (m6A), have emerged as crucial regulators of RNA metabolism and gene expression. These modifications can influence RNA stability, splicing and translation efficiency in both host and virus [5,8]. For example, m6A modifications can signal for either RNA degradation or stabilization depending on the binding proteins involved [9]. In the cytoplasm, m6A-modified RNAs are often recognized by YTHDF2 proteins to promote rapid mRNA decay. Conversely, the IGF2BP family of m6A readers can bind to the same modification and enhance RNA stability, protecting transcripts from degradation. This regulatory complexity has been demonstrated in RNA viruses such as HIV-1 [10], where m6A modifications on viral RNA interact with the nuclear reader YTHDC1 to control alternative splicing of viral transcripts and the cytoplasmic reader YTHDF2 to stabilize viral RNAs and enhance expression [10]. Moreover, depletion of m6A writers (e.g. METTL3/14) or overexpression of the m6A eraser ALKBH5 significantly reduced HIV-1 RNA and protein levels, confirming m6A’s essential role in the viral life cycle [10]. The landscape of RNA modifications during SARS-CoV-2 infection and their potential role in viral replication and host response represents an exciting area for new discoveries. Studying these modifications in the context of viral infections could reveal novel insights into host–virus dynamics and potential therapeutic targets.

In addition to RNA modifications, viral infections often trigger other complex changes in host gene expression, including alternative splicing and isoform variation. These changes can have profound implications for cellular response mechanisms, viral replication and pathogenesis [11,12]. Direct RNA sequencing using Oxford Nanopore Technologies (ONT) offers unique advantages for studying RNA biology, allowing both detection of RNA modifications and transcript isoforms in their native state [13]. The long reads generated by direct RNA sequencing provide a more robust platform for detecting alternative splicing events and isoform variations compared to short-read sequencing technologies, enabling a comprehensive view of transcriptome complexity during viral infection.

Previously, direct RNA sequencing has been used to study SARS-CoV-2 and SARS-CoV-2-infected cells using both VERO cells and directly from clinical samples [14,16]. Using different cell types can provide broader insights into RNA biology, as cell-specific factors may influence the viral lifecycle and host response. In this study, we used direct RNA sequencing on HEK cells infected with the SARS-CoV-2 Finland/1/2020 strain to comprehensively profile m6A RNA modifications, gene expression and RNA isoform dynamics. We focused on m6A modification specifically because it is the most extensively characterized RNA modification with established detection models compatible with ONT direct RNA sequencing. Our study identifies differentially modified m6A sites between mock and SARS-CoV-2-infected cells, uncovers novel RNA isoforms (alternative transcript variants) associated with viral infection and highlights pathways significantly impacted by these transcriptional changes. By integrating RNA modification prediction, isoform discovery and differential gene expression analysis, this work aims to expand our understanding of how RNA modifications and transcript diversity contribute to the host response to viral infection.

## Methods

### Cells and viruses

SARS‐CoV-2 isolate Finland/1/2020 (Genbank accession number MT020781.2) was grown, purified and titered as described previously [1]. HEK cells expressing Ace2 and TMPRSS2 (HEK-AT [17]) were maintained in Dulbecco’s Modified Eagle’s Medium (Sigma‐Aldrich) supplemented with 10% FBS (Gibco), 2 mM Glutamax (Gibco), 100 units of penicillin and 0.1 mg ml^−1^ streptomycin (PenStrep, Sigma‐Aldrich) and 1× non‐essential amino acids (NEAA) (Sigma‐Aldrich) (growth medium). Cells were incubated at 37 °C and 5% CO_2_ and passaged at 1:8 dilution every 5 days.

### Infection assays

For gene expression profiling, HEK-AT was grown to 90% confluency in the growth medium. At the time of infection, cells were washed twice with PBS and infection medium [MEM supplemented with 0.2% bovine serum albumin, 2 mM Glutamax (Gibco), 100 units of penicillin and 0.1 mg ml^−1^ streptomycin (PenStrep, Sigma‐Aldrich), 1× NEAA (Sigma‐Aldrich)]. The virus was added to the cells at a multiplicity of infection of 10 and incubated at 37 °C and 5% CO_2_. At 0 or 8 h post-infection (h.p.i), the medium was removed, and cells were washed twice with PBS and lysed using RA1 buffer (MACHEREY-NAGEL) supplemented with 20 mM dithiothreitol (DTT). RNA was purified using a NucleoSpin RNA Mini Kit (MACHEREY-NAGEL) according to the manufacturer’s instructions. All experiments were performed in triplicate, with three independent biological replicates for both SARS-CoV-2-infected and mock conditions.

### RNA extraction and reverse transcription

Total RNA was extracted using a NucleoSpin RNA Purification Kit (MACHEREY-NAGEL). RNA concentrations were measured with a Qubit RNA HS Kit (Invitrogen), and the quality was assessed with an Agilent 2100 BioAnalyzer and RNA 6000 Nano Kit. cDNA was synthesized in a 20 ul reaction containing 1 µg of total RNA, 5 µM of random hexamer primers (Thermo Scientific), 0.5 mM dNTP mix, 5 mM DTT, 2 units of Ribolock RNAse inhibitor (Thermo Scientific) and 200 units of SuperScript™ III Reverse Transcriptase (Invitrogen) in First-Strand Buffer (Invitrogen). RNA was incubated with primers for 5 min at 65 °C, after which the rest of the reaction components were added and reverse transcription was performed at 50 °C for 60 min. The reaction was stopped by heating at 70 °C for 15 min.

### Direct RNA sequencing

Total RNA samples extracted from six HEK-AT samples (MOCK 1–3 and infected 8 h 1–3) were sequenced using Oxford Nanopore PromethION 2 Solo with a Direct RNA Sequencing Kit (SQK-RNA002) according to the manufacturer’s instructions, except that the RNA Control Strand was diluted 1:20 before use. The amount of samples had a range between 83 and 155 ng of reverse-transcribed and adapter-ligated RNA that were sequenced with FLO-MIN106 flow cells using MinKNOW software v19.06.08 (Oxford Nanopore Technologies).

### Basecalling

Basecalling was performed using Dorado v4.3 software (https://github.com/nanoporetech/dorado) and the ‘rna002_70bps_hac@v3’ basecalling model. The ‘--estimate-poly-a’ option was used within Dorado to obtain poly(A) tail length predictions for each read.

### Identification and classification of sgRNA junctions in SARS-CoV-2

Our primary goal was to locate the unique junction point within each subgenomic RNA (sgRNA) where the leader sequence is joined to a downstream gene body. Using a method adapted from a similar approach described previously [18], we identified these junctions by analysing high-confidence alignments (MAPQ ≥20) and parsing their CIGAR strings (from minimap2 mapped bam files) for a skipped reference region (‘N’ operation) of at least 1,000 nt. To ensure each read represented a single transcript, only the first such junction from the 5′ end of a read was considered. For each valid junction, we recorded its 5′ donor and 3′ acceptor coordinates. These junctions were then classified as canonical only if the donor site occurred within 20 nt of the canonical TRS-L (position 69) and the acceptor site occurred within 15 nt of a known TRS-B site. Any junction failing to meet both of these criteria was classified as non-canonical.

### Genome coverage depth

Sequencing reads were aligned to a reference genome comprising the human genome (GRCh38) and the SARS-CoV-2 reference genome (GenBank accession: NC_045512.2) using minimap2 v2.26 [19] with the suggested Nanopore Direct RNA-seq settings (-ax splice -uf -k14). To assess SARS-CoV-2 genome coverage, we used SAMtools v1.12 [20] depth command with no upper limit on per-position depth (-d 0). This analysis generated base-by-base coverage depth across the viral genome.

### *De novo* m6A modification prediction

While Dorado v4.3 offers basecalling functionality, it lacks a dedicated model for identifying m6A modifications within RNA sequences generated from the RNA002 direct RNA library preparation. To overcome this limitation and predict potential m6A modification sites, we used a separate tool for *de novo* m6A modification prediction.

We utilized a modification-aware m6A basecalling model developed by Cruciani *et al.* [21]. This model, retrieved from the public GitHub repository https://github.com/novoalab/m6ABasecaller/blob/main/basecalling_model/ (accessed January 2024), incorporates information about potential m6A modifications during the basecalling process. This allows for the identification of putative m6A sites within the RNA transcripts.

We used modPhred v3.6.1 [22] for the analysis. The reference genome used within modPhred was a combined human genome (GRCh38) and SARS-CoV-2 genome (NC_045512.2) to account for both host and potential viral RNA. The fast5 files from PromethiON 2 Solo were used as an input for the modPhred run. The *de novo* m6A modification prediction was run separately for each sample (three infected and three mock replicate samples). To enhance confidence in predicted m6A sites, we only considered modifications consistently observed across all three replicates within each sample group (infected or mock).

To investigate sequence motifs associated with predicted RNA modifications in the SARS-CoV-2 genome, we analysed the nucleotide sequence surrounding each identified modified site. For each modified site, we extracted the flanking sequence comprising 10 nucleotides upstream and 10 nucleotides downstream, resulting in a 21-nt window centred on the modified base. The resulting set of 21-nt flanking sequences was compiled into a FASTA file. This file was then uploaded to the MEME Suite web server (https://meme-suite.org) [23] for *de novo* motif discovery. MEME was run using default parameters.

To identify individual reads containing m6A modifications, we used a custom Python script to filter the ‘fastm’ files generated by modPhred. A read was considered to contain a modification if at least one base showed a modification probability greater than 0.5. This probability is derived from the quality score line in the fastm record.

### Differential RNA modifications in mock and infected cells in humans

To identify differentially modified RNA sites between mock and infected cells, we utilized the xPore v2.1 tool [24]. First, direct RNA-seq reads were aligned to the human reference transcriptome (GRCh38) using minimap2 [19] with options ‘-ax splice -uf -k14’. The nanopolish eventalign module [25] was used to generate the necessary event alignment file. We used the ‘--scale-events --signal-index’ options during this step. The created event alignment files were then used with the ‘xpore dataprep’ command with human reference transcriptome Fasta and GTF annotation files. Differential RNA modifications between mock and infected samples were identified using the ‘xpore diffmod’ command. This analysis included six samples (three mock replicates: mock1, mock2, mock3; and three infected replicates: 8h1, 8h2, 8h3). We used pre-filtering criteria within ‘xpore diffmod’ using a t-test with a 0.05 significance threshold and a minimum read count of 15 (‘readcount_min: 15’). Differential modification sites were filtered using ‘xpore postprocessing’ default settings to retain only those sites whose modification directionality ('higher' or 'lower') agreed with the majority direction within their respective 5-mer context, as determined by xPore. To further enhance confidence in the identified modifications, we manually filtered the results. We only retained sites with a differential modification rate greater than 0.5 and a *P*-value less than 0.001. Finally, we excluded any sites with missing coverage in any of the replicates to ensure reliable data.

### Gene enrichment analysis

We performed gene enrichment analysis using the Enrichr web application [26]. For pathway enrichment, we focused on the Kyoto Encyclopedia of Genes and Genomes (KEGG) 2021 Human Pathway dataset, while Gene Ontology (GO) terms were used for ontology enrichment, covering biological processes, cellular components and molecular functions. We used an adjusted p-value<=0.05 as a significance threshold.

### Isoform discovery and quantification

Direct RNA sequencing reads were first aligned to the human reference genome (GRCh38) in FASTA format using minimap2 [19]. The options ‘-ax splice -uf -k14’ were used. Following alignment, all generated BAM files were used for isoform discovery with the Bambu v3.2.4 tool [27]. This tool used the provided reference genome annotations in GTF format (GRCh38) to reconstruct full-length transcripts from the aligned reads.

Bambu not only facilitates isoform discovery but also provides quantification information for the identified isoforms. From several different quantification outputs, we selected ‘fullLengthCounts_transcript.txt’ for downstream analysis related to transcript expression. This file contains estimates of read counts mapped as full-length reads for each transcript. To identify differentially expressed transcripts, transcripts quantified in ‘fullLengthCounts_transcript.txt’ were analysed using DESeq2 [28]. A significance threshold of adjusted *P*-value<0.05 was applied. In addition, we used the ‘counts_gene.txt’ output file from the Bambu tool for gene expression analysis. To identify isoform switching and alternative splicing between mock and infected cells, we used the IsoformSwitchAnalyzeR R package [29].

## Results

### Mapped reads

**Fig. 1.**
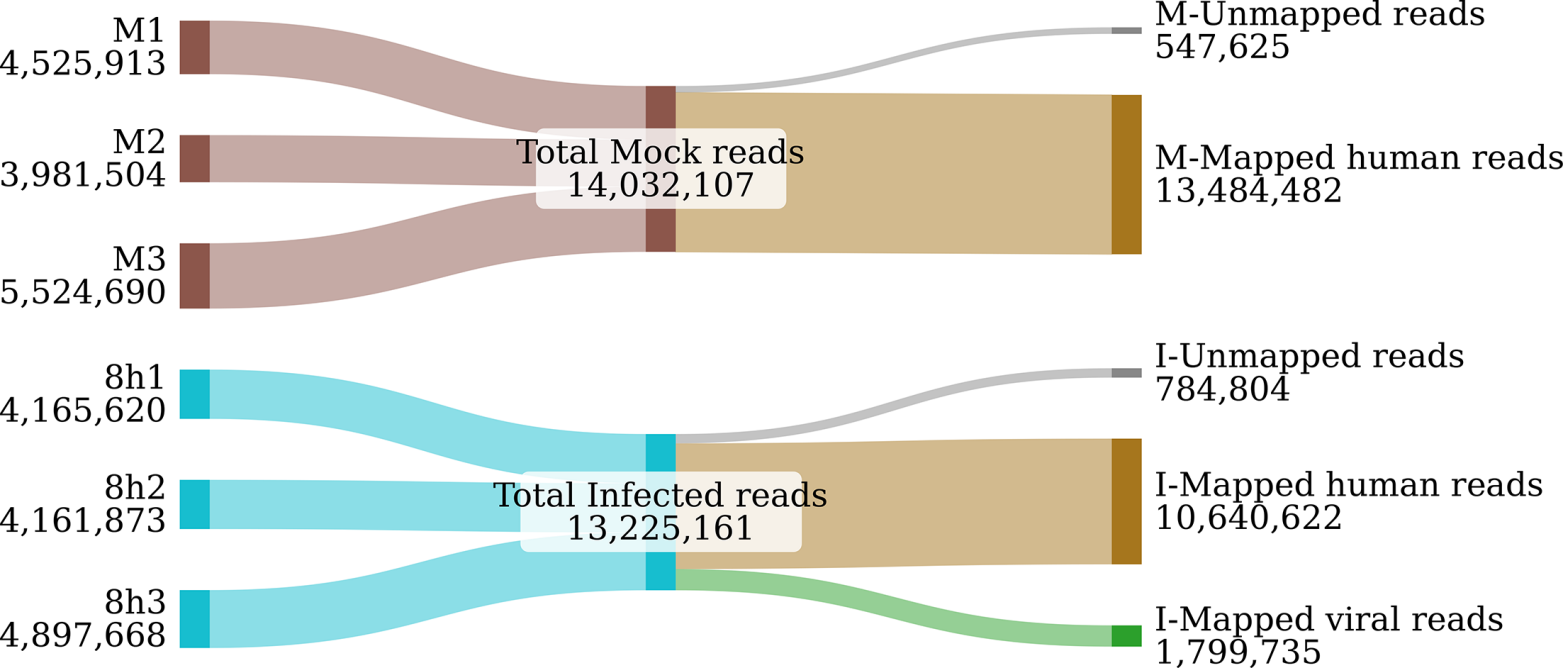
Sankey diagram illustrating the distribution of direct RNA sequencing reads. The left side displays the read counts for each sample, while the centre shows the combined total from all three replicates. The right side represents the total number of mapped reads, summed across all triplicates.

In this study, native RNA from mock human cells and SARS-CoV-2-infected cells was sequenced using the ONT PromethION 2 Solo platform using a Direct RNA Sequencing Kit (SQK-RNA002). The number of mapped reads in the mock samples ranged from 3.85×10^6^ to 5.25×10^6^, with all reads mapping to the human genome. In contrast, for SARS-CoV-2-infected samples, the mapped read numbers ranged from 3.93×10^6^ to 4.53×10^6^, with 568,707 to 642,725 (14.2%–14.8%) reads mapping to the viral genome. Analysis of mapped read lengths revealed a median length between 668 and 781 bp for mock samples, with a maximum read length of up to 9,905 bp. In the infected samples, the median read length was higher, ranging from 757 to 819 bp, and the maximum read lengths reached up to 17,021 bp (Fig. 1 and Table S1, available in the online Supplementary Material). The poly(A) tail length prediction in SARS-CoV-2 direct RNA reads has a median length of 63 nt poly(A) tail (Fig. S1).

### Viral transcriptome

**Fig. 2.**
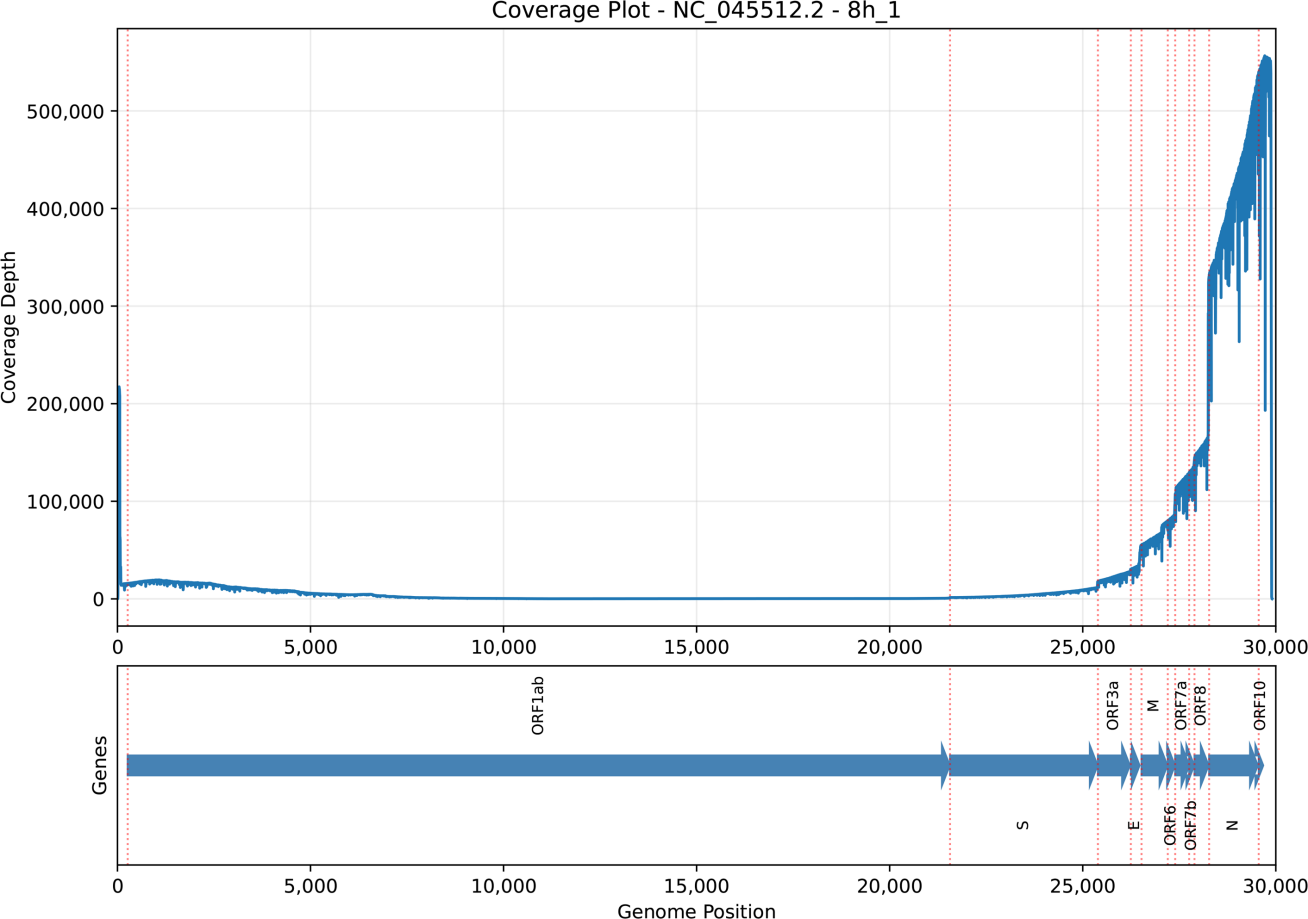
Genome coverage depth and gene annotations in SARS-CoV-2 in sample 8h1. The upper panel displays the sequencing coverage depth across the genomic positions, represented by blue markers. Red dotted lines indicate the start positions of genes along the genome. The lower panel illustrates the gene architecture using arrows, with each arrow representing a gene and indicating its position and transcriptional direction.

Our direct RNA sequencing data revealed how SARS-CoV-2 produces its transcriptome during infection. We observed an uneven pattern of sequencing coverage depth across the viral genome. While most of the genome showed relatively low coverage depth (1,000–20,000×), we saw a dramatic increase in coverage depth toward the 3′ end, reaching up to 500,000× (Figs 2 and S2). The highest read coverage appears at the nucleocapsid (*N*) and *ORF10* gene region (Figs 2 and S2).

The asymmetric coverage pattern reflects the canonical coronavirus transcription strategy, where discontinuous transcription generates nested sgRNAs sharing a common 3′ end. Consistent with this, the observed jumps in coverage depth aligned precisely with the start positions of viral genes, each jump marking the beginning of a new subgenomic RNA. SARS-CoV-2 sgRNA profiling revealed that sgRNA transcripts were highly abundant, forming an average of 37.7% of all sequencing reads across three biological replicates. Canonical sgRNAs dominated, containing 85.9%±0.7% (mean±sd) of all junctions, while non-canonical sgRNAs (nc-sgRNAs) formed 14.1%±0.7%. Among canonical sgRNAs, the *N* gene consistently dominated, accounting for 73.6–75.6% of canonical junctions. Other abundant transcripts included ORF7a (9.5–9.6%), M (5.8–6.4%) and ORF8 (3.6–3.9%). Low levels of canonical sgRNAs for ORF6, ORF3a, E and S were detected in all samples. ORF10 was rarely detected, representing <0.01% of canonical junctions (Table 1).

**Table 1.** Summary of sequencing reads that mapped to the SARS-CoV-2 genome, junction classifications and canonical sgRNA distribution across three biological replicates

	8h1	8h2	8h3	Mean
Total Sars-Cov-2 Reads	568,707	588,303	642,725	599,911.7
Total junctions (% of total)	218,737 (38.46%)	228,588 (38.86%)	230,828 (35.91%)	226,051 (37.74%)
Canonical junctions (%)	189,552 (86.7%)	195,366 (85.5%)	197,198 (85.4%)	194,038.7 (85.9%)
Non-canonical junctions (%)	29,185 (13.3%)	33,222 (14.5%)	33,630 (14.6%)	32,012.3 (14.1%)
Canonical reads per gene (% of canonical junctions)				
N	73.60%	73.40%	72.60%	73.20%
ORF7a	9.90%	9.40%	9.60%	9.60%
M	5.80%	6.00%	6.40%	6.10%
ORF8	3.70%	3.90%	3.80%	3.80%
ORF6	3.10%	3.20%	3.40%	3.20%
ORF3a	2.50%	2.60%	2.80%	2.60%
E	1.10%	1.20%	1.20%	1.20%
S	0.26%	0.30%	0.29%	0.28%
ORF10	<0.01%	<0.01%	<0.01%	<0.01%

### *De novo* m6A modification prediction

Our *de novo* m6A modification analysis predicted 16 m6A modification sites across the SARS-CoV-2 genome that were consistently detected in all three biological replicates of the infected samples. These modifications showed a non-random distribution across the viral genes, with the majority of the sites concentrated in two major viral genes: *ORF1ab* and spike (*S*) genes.

Specifically, eight m6A modifications were identified within the *ORF1ab* gene region, while seven modifications were located in the *S* gene. A single m6A modification site was detected in the *ORF3a* gene. Notably, no consistent m6A modifications were observed in other viral genes (Table 1 and Fig. S3).

The coverage depth for these modification sites varied considerably across the genome. The highest coverage was observed at position 25,987 (23,904–28,519 reads across replicates), while the lowest coverage was detected at position 13,921 (105–146 reads). The modification frequency (percentage of modified reads) ranged from 5% to 30% across different sites, with particularly high modification rates (27–30%) observed at position 16,754 across all three replicates (Table 2).

**Table 2.** m6A modification sites identified in the SARS-CoV-2 genome across three biological replicates of infected samples at 8 h.p.i.

Position	mod	ORF	Coverage 8h1	% mod 8h1	Coverage 8h2	% mod 8h2	Coverage 8h3	% mod 8h3
2001	m6A	*ORF1ab*	15,682	7	16548	7	18,074	10
3549	m6A	*ORF1ab*	8,641	7	9276	6	9,264	9
4818	m6A	*ORF1ab*	6,059	7	6447	6	6,725	8
10602	m6A	*ORF1ab*	241	9	285	8	304	9
13921	m6A	*ORF1ab*	105	9	146	10	123	21
14420	m6A	*ORF1ab*	131	12	131	15	144	8
15846	m6A	*ORF1ab*	155	10	112	7	139	17
16754	m6A	ORF1ab	128	27	92	30	122	30
21719	m6A	*S*	1,398	6	1,539	6	1,874	8
21778	m6A	*S*	1,347	17	1,452	18	1,846	23
22094	m6A	*S*	1,585	8	1,708	7	2,187	8
23264	m6A	*S*	3,162	9	3,249	8	4,211	10
23302	m6A	*S*	2,627	7	2,715	7	3,539	10
24220	m6A	*S*	5,750	6	6,076	5	7,315	7
24666	m6A	*S*	6,904	7	7,422	7	8,825	7
25987	m6A	*ORF3a*	23,904	6	25,893	6	28,519	8

We performed a *de novo* motif discovery analysis to identify any recurring sequence patterns within the 21-nt regions flanking the m6A modification sites. Our analysis did not show a statistically significant, highly conserved motif. Nevertheless, we observed a low-confidence, recurrent ‘TGGACC’-like motif associated with the modified sites.

With *de novo* m6A modification prediction analysis in the human genome, we identified 10,411 modified positions in infected cells and 13,031 modified positions in mock cells, of which 9,256 were observed in all samples, regardless of infection status (Table S2). To ensure reliability, we only considered a position as modified if all three replicates within each condition showed a modification signal.

The table shows all m6A modification sites consistently detected across three biological replicates (8h1, 8h2 and 8h3) in SARS-CoV-2-infected samples. Position indicates the nucleotide position in the SARS-CoV-2 genome (NC_045512.2). Mod denotes the type of RNA modification detected. The ORF column indicates the ORF corresponding to each base position. Coverage represents the total number of reads covering each position in each replicate. % mod indicates the percentage of reads showing m6A modification at each position. All modifications were identified using modPhred v3.6.1 with a combined human (GRCh38) and SARS-CoV-2 reference genome.

We also performed read-level analysis to quantify the proportion of individual SARS-CoV-2 reads containing m6A modifications across three biological replicates. A read was classified as modified if at least one nucleotide position exhibited a modification probability exceeding 0.5 (see the ‘Methods’ section). We saw that SARS-CoV-2 reads contained m6A modifications ranging from 33.00% to 49.25% (mean=40.23%). Importantly, we observed consistently higher modification rates in sgRNA reads compared to gRNA reads across all three replicates. The sgRNA modification frequencies ranged from 36.09% to 53.01% (mean=43.72%), while gRNA sequences showed modification rates of 31.03%–47.13% (mean=38.11%). Statistical analysis using a paired t-test confirmed this difference was significant (*P*=0.0024), indicating that sgRNA transcripts are preferentially targeted for m6A modification compared to gRNA reads.

### Differential RNA modifications in mock and infected cells in humans

Differential RNA modification analysis between mock and infected cells showed significant changes at 254 positions across the transcriptome. Of these, 119 positions showed significantly lower modification rates in infected cells compared to mock cells, distributed across 118 unique transcripts corresponding to 83 distinct genes. On the contrary, 135 positions showed significantly higher modification rates in infected cells, distributed across 134 unique transcripts associated with 95 genes (Table S3).

Pathway enrichment analysis of the 83 genes with reduced modification rates in infected cells revealed significant enrichment in one KEGG pathway. The neurotrophin signalling pathway showed significant enrichment (adjusted *P*-value=0.0158), involving five genes: RPS6KA3, IRAK1, MAGED1, MATK and PSEN2. Gene Ontology (GO) analysis identified significant enrichment in multiple categories. Two biological process terms showed significant enrichment: DNA-templated DNA replication maintenance of fidelity (GO:0045005, adjusted *P*-value=0.01383) and replication fork processing (GO:0031297, adjusted *P*-value=0.01383), both involving the same set of genes (EXD2, MMS22L, FANCM and RFWD3) (Table S3).

In contrast, genes with increased modification rates in infected cells (*n*=95) did not show significant enrichment in either KEGG pathways or GO terms. This lack of enrichment suggests that these modifications may affect a more diverse set of cellular processes rather than concentrating on specific pathways. However, despite the absence of statistically significant enrichment, we observed four genes (IRF3, MAP3K7, GNAI1 and PTK2) associated with the KEGG pathway ‘Human immunodeficiency virus 1 infection’.

### Isoform discovery and differential gene/transcript expression

Isoform reconstruction using the Bambu tool [27] identified a total of 206,990 transcripts in the human genome. Of these, 206,601 (99.8%) were previously annotated, while 389 (0.2%) were novel. The novel transcripts were distributed across all chromosomes, including mitochondria and small contigs such as GL000009.2, GL000194.1, GL000195.1, GL000205.2 and GL000218.1. In the viral genome, Bambu identified only three transcripts. However, these transcripts spanned nearly the entire length of the genome, which raised concerns about their accuracy. Given the well-characterized nature of the SARS-CoV-2 genome, which is known to produce a larger and more diverse set of transcripts, these predictions appeared inconsistent with expectations. Therefore, we suspect these transcripts might represent false positives, potentially due to limitations in the isoform prediction for highly compact viral genomes.

Differential gene expression analysis, based on Bambu gene quantification data, was performed on a total of 58,776 genes (58,735 Ensembl-annotated genes and 41 novel genes identified during isoform discovery with Bambu). Among these, 24,440 genes had non-zero read counts and were tested in DESeq2. DESeq2’s internal independent filtering step automatically flagged 19,739 genes (81%) as low-count features, leaving 4,701 genes for statistical testing. We identified 28 genes significantly upregulated and 56 genes significantly downregulated in infected cells (Fig. S4 and Table S4). Enrichment analysis of the 28 upregulated genes showed significant enrichment in the KEGG pathway ‘Ribosome biogenesis in eukaryotes’ (adjusted *P*-value=0.004), involving three genes: SNORD3A, RNA5-8SN5 and RMRP. While no GO terms were found to be significantly enriched in these upregulated genes, the upregulation of GRSF1 is noteworthy due to its association with infectious disease pathways [30].

**Fig. 3.**
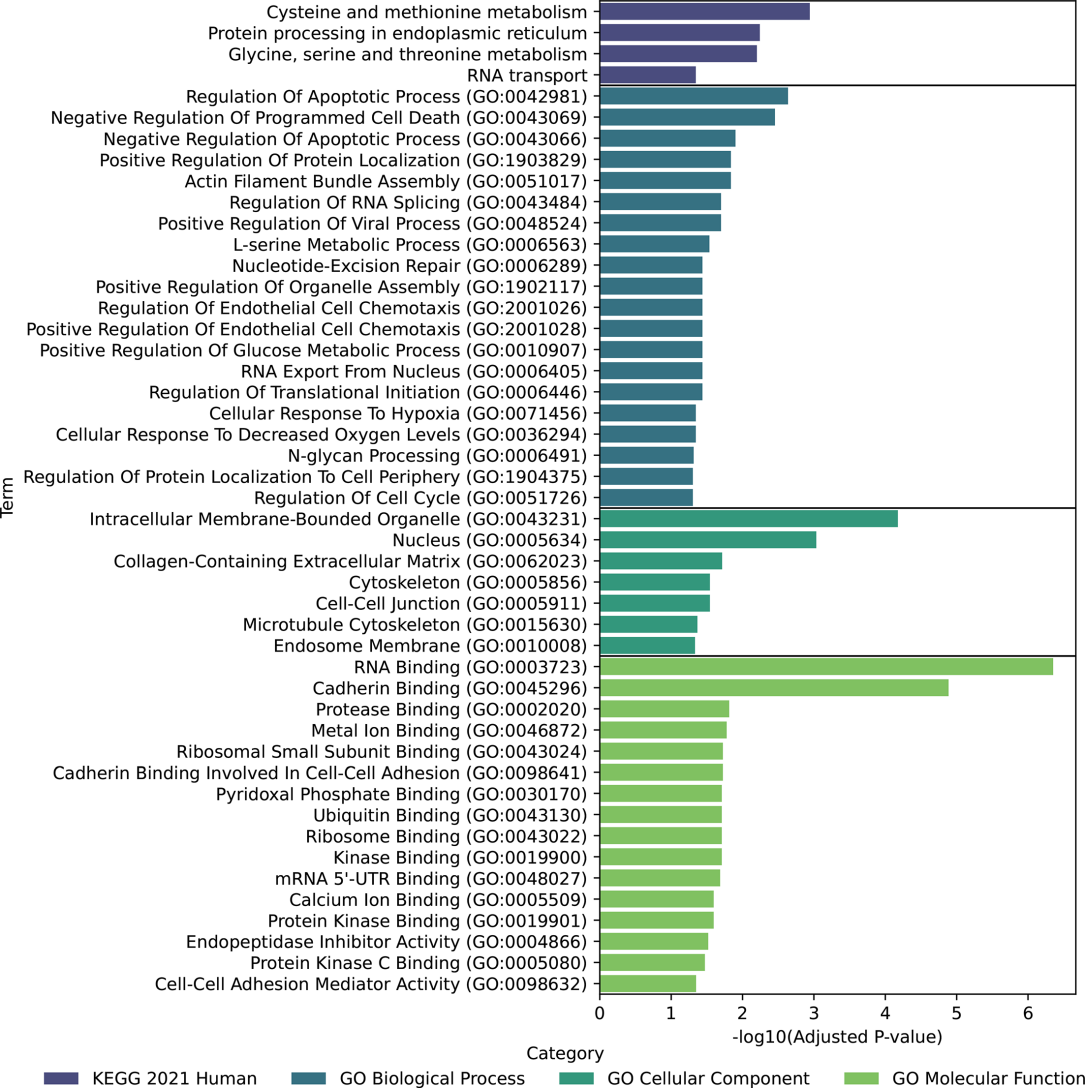
The bar chart displays significantly enriched terms for downregulated genes in infected cells. Each colour represents a different category. The *x*-axis shows the -log10 of the adjusted *P*-value.

The 56 downregulated genes in infected cells showed more extensive enrichment across multiple categories. In terms of KEGG pathways, we observed enrichment in cysteine and methionine metabolism, as well as protein processing in the endoplasmic reticulum. GO biological process analysis revealed enrichment in regulation of apoptotic process (GO:0042981) and negative regulation of programmed cell death (GO:0043069). GO cellular component analysis showed enrichment in intracellular membrane-bounded organelle (GO:0043231) and nucleus (GO:0005634). Finally, GO molecular function analysis indicated enrichment in RNA binding (GO:0003723) and cadherin binding (GO:0045296). A comprehensive list of enriched terms is presented in Fig. 3 and Table S4c.

Differential transcript expression analysis, based on Bambu transcript quantification data, revealed additional insights into the transcriptional landscape during infection. In total, our transcript count matrix contained 206,990 transcripts (206,601 Ensembl-annotated transcripts and 389 novel isoforms identified by Bambu). Of these, 33,162 transcripts had non-zero read counts and were tested with DESeq2. DESeq2’s internal independent filtering automatically removed 21,415 transcripts (65%) as low-count features, leaving 11,747 transcripts for statistical testing. We identified 19 transcripts (corresponding to 19 genes) significantly upregulated in infected cells (Fig. S5 and Table S5a). Interestingly, only three (ENSG00000197903, HIST1H2BK; ENSG00000117318, ID3; ENSG00000269900, RMRP) of these 19 genes were also identified as significantly upregulated in the gene-level analysis, indicating that transcript-level differences occur during infection in addition to gene-level changes. Enrichment analysis of these 19 upregulated transcripts revealed enrichment in the KEGG pathway ‘Coronavirus disease’ (involving *RPL23*, *RPSA* and *RPS27A* genes) and ‘*Vibrio cholerae* infection’ (involving *GNAS* and *ACTG1* genes). Notably, differential expression of these genes was not observed in the gene quantification data, suggesting that specific transcripts, rather than overall gene expression, were upregulated in infected cells.

Among the 19 significantly upregulated transcripts in infected cells, we identified a novel transcript, BambuTx284, corresponding to the gene ENSG00000197903 (HIST1H2BK). This transcript shares the same protein-coding sequence as the reference transcript ENST00000356950 but exhibits an extended 3′ region supported by RNA-seq reads (Fig. S6). Although the additional sequence in BambuTx284 does not contribute to protein coding, it may represent an untranslated region or potentially serve as a regulatory element influencing transcript stability, localization or translational efficiency. This discovery suggests that ENSG00000197903 may produce multiple isoforms, including both coding and non-coding regions, with the novel isoform BambuTx284 being statistically significantly more expressed in infected cells.

Additionally, we identified 12 transcripts (corresponding to 12 genes) significantly downregulated in infected cells (Table S5b). Of these, five genes were also identified as significantly downregulated in the gene-level analysis. Enrichment analysis of these 12 genes revealed only 2 enriched KEGG terms: cysteine and methionine metabolism and amyotrophic lateral sclerosis. No significant GO term enrichments were observed for these downregulated transcripts.

### Isoform switching and alternative splicing

Using the IsoformSwitchAnalyzeR package, we analysed differential isoform usage in mock and SARS-CoV-2-infected human cells. A total of 37 isoforms were identified as differentially expressed between the 2 conditions, corresponding to 24 significant isoform switches across 21 genes (Fig. S7 and Table S6a). Among the 37 significant isoforms that contributed to the isoform switching in 21 genes, a total of 48 alternative splicing events were detected. These included four exon skipping (ES) events, three intron retention (IR) events, five alternative 5′ splice site (A5) events and two alternative 3′ splice site (A3) events. Additionally, there were 19 alternative transcription start site (ATSS) events and 15 alternative transcription termination site (ATTS) events. No mutually exclusive exon or multi-exon skipping events were observed (Table S6b). Enrichment analysis revealed significant enrichment in pathways and processes linked to infection and mitochondrial function (Table S6c). Genes involved in thermogenesis (GNAS, ATP5MC3 and COX6B1; adjusted *P*=0.007) and platelet aggregation (GNAS, ACTG1 and HSPB1; adjusted *P*=0.0016) were enriched, reflecting potential alterations in cellular metabolism and host defence mechanisms. Notably, infection-related pathways such as amoebiasis and *Vibrio cholerae* infection (e.g. GNAS, ACTG1 and HSPB1; adjusted *P*<0.01) were also identified. Mitochondrial components, including the inner and outer membranes, were highly enriched (e.g. ATP5MC3, COX6B1 and SLC25A38; adjusted *P*<0.01), underscoring the role of mitochondrial reprogramming during viral infection.

## Discussion

In this study, we used direct RNA sequencing to comprehensively profile RNA modifications and transcriptional changes in human HEK cells during SARS-CoV-2 infection. Our analysis provided new insights into RNA modifications and transcriptional dynamics in SARS-CoV-2-infected human cells, advancing our understanding of how viral infections influence host and viral RNA landscapes. Moreover, we were able to study RNA modifications and polyadenylation in SARS-CoV-2.

Long-read direct RNA sequencing is an emerging technology that enables a comprehensive study of RNA modifications in their native context [13]. In this study, we utilized the Oxford Nanopore PromethION 2 Solo platform with the SQK-RNA002 direct RNA sequencing kit to generate direct RNA sequencing data. While Oxford Nanopore software provides basecalling functionality, it lacks a dedicated model for identifying modifications in RNA sequences generated from the SQK-RNA002 protocol. To address this limitation, we utilized a recently developed m6A modification prediction model by Cruciani *et al.* [21] [21]. It is important to acknowledge that newer sequencing chemistries, particularly the RNA004 kit, have demonstrated significant improvements in RNA modification detection accuracy and sensitivity compared to RNA002 [31]. However, while at least one study has utilized RNA004 chemistry for SARS-CoV-2 sequencing [32], no studies have yet reported RNA modification analysis from SARS-CoV-2 using this newer chemistry, making direct performance comparisons in this viral context unavailable. Our analysis revealed 16 m6A modification sites within SARS-CoV-2 transcripts, predominantly located in the *ORF1ab* and *S* genes. The concentration of modifications in these regions is particularly noteworthy, as *ORF1ab* encodes the viral replicase machinery and *S* is crucial for host cell entry [33]. This distribution suggests that m6A modifications may play an important role in regulating key viral processes, potentially influencing both viral replication and the efficiency of host cell infection. Interestingly, our findings differ from previous studies of SARS-CoV-2 RNA modifications. We identified a completely different set of sites compared to the 41 locations previously reported using long direct RNA sequencing [14]. Similarly, another study using the m6anet tool for modification prediction identified 15 modified sites [15], which also showed no overlap with our findings. These differences can be caused by several key methodological differences. First, our analysis focused on modifications at 8 h.p.i., whereas previous studies examined modifications at 24 and 48 h.p.i. [14,15]. Second, we used the Finland/1/2020 strain, while other studies used different viral strains with potential single-nucleotide variations [14,15]. Third, our use of HEK cells contrasts with the Vero cells used in previous studies [14,15]. Finally, our computational approach using the Cruciani *et al*. model [21] differs from both the *in vitro* transcribed control sequence comparison method used in the first study [14] and the m6anet tool used in the second study [15]. Beyond direct RNA sequencing approaches, studies using alternative methods such as miCLIP and meRIP-seq have also reported distinct modification patterns, identifying 8 and 16 m6A positions, respectively [34,35], which showed no overlap with our results. Similarly, another study using meRIP-seq [36] detected m6A peaks within the *N* gene region, and a separate study using m6A RIP-seq [37] reported 10 m6A modifications mainly in the N/ORF10 region; none of these positions overlapped with our findings. The different modification patterns observed across different time points and experimental conditions suggest that SARS-CoV-2 may use dynamic epitranscriptomic regulation, adapting its RNA modification profile as infection progresses from early establishment to mature infection. These variations in experimental design and methodology underscore the importance of considering temporal dynamics, cell types and analytical approaches when studying viral RNA modifications.

Interestingly, a recent study conducted a detailed m6A modiﬁcation analysis using 2,190,667 SARS-CoV-2 sequences from 166 countries and reported 51 m6A modification sites across the viral genome [38]. Two reported modification sites, 13,922 and 25,988, are only one base different from our reported m6A modification positions (13,921, 25,987), a difference likely caused by viral variant differences. Hence, this large-scale analysis provides strong support for the modification of positions 13,921 and 25,987. Although the study [38] does not report a link between these positions and viral pathogenicity, their independent identification suggests these modifications may be functionally important.

Previous studies have reported an enrichment of an AAGAA-like motif flanking m6a modification sites in the SARS-CoV-2 genome [14]. In our analysis, we examined the 21-nt regions surrounding the identified m6A sites but did not detect a statistically significant or highly conserved motif. However, we observed a low-confidence, recurrent ‘TGGACC’-like sequence associated with the modified regions. The discrepancy between our findings and the earlier report may be explained by the fact that our predicted modification sites differ from those previously identified. This lack of motif conservation could suggest variability in m6A site selection depending on experimental conditions, viral strain, host cell type or methodological differences. It is also possible that m6A occurrence in SARS-CoV-2 is not strictly dependent on a conserved sequence motif but may be influenced by secondary RNA structures or interactions with host RNA-binding proteins.

Our findings on SARS-CoV-2 transcription align with the canonical coronavirus discontinuous transcription mechanism described by Malone *et al*. [39]. The observed uneven sequencing coverage pattern, with dramatic increases towards the 3′ end, reflects the production of nested subgenomic RNAs (sgRNAs) sharing a common 3′ end. Sharp increases in coverage depth at viral gene start sites further corroborate this model, indicating the initiation points of new sgRNAs. To evaluate gene expression levels accurately, we focused on quantifying sgRNAs, rather than relying solely on total genome coverage, which can be skewed by genomic RNA (gRNA) accumulation. The highest total read coverage was observed at the *N* and *ORF10* gene regions, suggesting significant transcriptional activity in these areas. When focusing on sgRNA abundance, the *N* gene had the highest number of sgRNA reads. This observation aligns with previous studies reporting elevated *N* gene transcription and the corresponding abundance of N protein in infected cells [40,41]. In contrast, while the *ORF10* gene region also showed high total mapped read coverage, our analysis revealed only a small number of canonical sgRNA reads associated with this gene. This inconsistency suggests that ORF10 transcription via sgRNAs is relatively low, and the elevated read counts in this region are likely related to gRNA rather than sgRNAs. This interpretation is in line with previous studies, which have reported very low or no sgRNAs for ORF10 [41,43].

Time after infection also impacts the composition of sgRNAs, particularly the proportion of nc-sgRNAs. Previous studies have shown that the abundance of nc-sgRNAs increases over time. Notably, it has been reported that nc-sgRNAs rose from ~15–19% at 4–5 h.p.i. to ~25–27% at 24 h [18], reaching up to 33% in some cell culture models [18]. In our dataset, collected at 8 h.p.i., nc-sgRNAs accounted for 14.1%±0.7% of all junctions, with canonical sgRNAs dominating. This relatively low proportion of non-canonical junctions may reflect an intermediate stage of infection. The accumulation of nc-sgRNAs may be a gradual process, potentially linked to the progression of viral replication.

Our read-level m6A analysis quantified the proportion of individual SARS-CoV-2 reads containing m6A modifications. We showed that both gRNA and sgRNA transcripts are subject to m6A modification. The occurrence of m6A on both transcript classes is consistent with earlier reports [44]. Our findings, however, showed a significant and novel distinction: a consistently higher proportion of m6A-modified reads in sgRNA transcripts (mean=43.72%) compared to gRNA reads (mean=38.11%). This statistically significant difference (*P*=0.0024) suggests that sgRNA transcripts are preferentially targeted for m6A modification.

Bovine coronavirus has been shown to exhibit changes in poly(A) tail length during infection, with lengths of ~45 nt observed at 0–2 h.p.i., increasing to ~65 nt at 4–12 h and shortening to ~40 nt at later stages [45]. Our samples, collected at 8 h.p.i., displayed an average poly(A) tail length of 63 nt, which is consistent with these earlier findings [45]. Similarly, a median poly(A) tail length of 47 nt has been reported at 24 h.p.i. in previous studies [14].

In addition to quantifying m6A-modified reads, we also examined the poly(A) tail lengths of modified versus non-modified SARS-CoV-2 transcripts. Previous studies have reported that m6A-modified reads tend to exhibit shorter poly(A) tails, which may influence RNA stability [14]. To investigate this in our dataset, we compared the poly(A) tail lengths between modified and non-modified reads across all transcripts, as well as within the canonical and non-canonical transcript subsets. However, we did not observe a statistically significant difference in poly(A) tail length between the groups (overall comparison *P*=0.9566; canonical transcripts *P*=0.5129; non-canonical transcripts *P*=0.4298).

Our RNA m6A modification prediction site analysis in human cells identified 10,411 m6A-modified sites in infected cells and 13,031 in mock cells. However, we noted that sequencing depth likely influences the detection of m6A modifications. The infected cell samples averaged 3.5×10^6^ human-mapped reads per sample, while mock cells averaged 4.4×10^6^, suggesting that the observed difference in m6A sites may partially reflect this variation in sequencing depth. This relationship between read depth and m6A site detection aligns with previous findings in HEK293T WT cells, where analysis of two replicates yielded 6,664 m6A sites from 1.2×10^6^ reads and 1,625 sites from 400K reads, respectively [21]. To account for these technical variations, we used a differential modification analysis method for long direct RNA sequencing data [24]. This analysis revealed 254 positions across the transcriptome with significant m6A modification differences between mock and infected cells. Previously, it has been reported that in viral infections, m6A often positively correlates with gene expression in immune response pathways, as immune response genes are more active with higher m6A levels, like IFIH1, TNFAIP3, IFIT1 and IFIT2 [46]. Here, we focused on both increased m6A modification rate sites and decreased m6A modification rate sites during the infection. Decreased m6A modification rate sites can be interesting too, since some genes with lower m6A modifications might become more stable or could be translated more efficiently [47]. It is also possible that the virus might be exploiting m6A modifications to modulate host gene expression, either to suppress immune response genes or alter other pathways favouring viral replication or persistence. For the genes that we saw significantly higher modification rate in infected cells, we did not see any significant enrichment, but there were interesting genes related to the KEGG pathway ‘Human immunodeficiency virus 1 infection’, such as IRF3, MAP3K7, GNAI1 and PTK2. Previously, it has been shown that IRF3 is a crucial component of the antiviral response pathway and requires nuclear translocation to function properly [48]. SARS-CoV-2 interferes with IRF3 and blocks IRF3’s nuclear translocation [48]. The increased m6A modification rate within the IRF3 transcript in this study might represent another layer of regulation in this immune evasion strategy of SARS-CoV-2. Moreover, we saw a significant modification rate increase in TRIM family genes such as TRIM16L and TRIM56. It has been shown that TRIM proteins play a role in immune response [49], and significant modification rate increase may show that infection of SARS-CoV-2 affects the immune gene epitranscriptomic regulations. An interesting observation from our study was a significant reduction in m6A modification levels in SARS-CoV-2-infected cells compared to mock-infected controls. This aligns with recent findings demonstrating that SARS-CoV-2 infection globally depletes cellular m6A RNA methylation by disrupting METTL3 localization and complex formation [37]. Particularly noteworthy was the decreased m6A modification of five genes (RPS6KA3, IRAK1, MAGED1, MATK and PSEN2) showing significant enrichment in the neurotrophin signalling pathway. This finding is interesting given the several evidence of neurological effects in coronavirus disease 2019 (COVID-19) patients [50]. The neurotrophin signalling pathway plays crucial roles in neuronal survival, differentiation and plasticity [51], and its dysregulation through altered m6A modification could potentially contribute to the neurological symptoms associated with SARS-CoV-2 infection. This finding suggests a mechanism by which SARS-CoV-2 could influence neurological outcomes through the modulation of host cell epitranscriptomic regulation. Future studies should investigate whether this decrease in m6A modification directly contributes to COVID-19-associated neurological symptoms and whether targeting these pathways could have therapeutic potential.

Differential expression analysis revealed complex patterns of host cell response, with distinct signatures at both gene and transcript levels. Significantly upregulated genes in infected cells enriched in ribosome biogenesis in eukaryotes KEGG pathway, suggesting that SARS-CoV-2 may manipulate host cell translational machinery to facilitate viral protein production.

Beyond differential gene expression, our isoform switching analysis uncovered transcriptomic remodelling in SARS-CoV-2-infected human cells. We identified 24 significant isoform switches spanning 21 genes, coupled with 48 alternative splicing events. Pathway enrichment analysis implicated mitochondrial reprogramming and immune-related pathways, such as thermogenesis and platelet aggregation, suggesting adaptive metabolic and host defence responses during infection. Notably, enrichment of mitochondrial genes, including ATP5MC3 and COX6B1, points to potential viral manipulation of mitochondria. This aligns with previous studies demonstrating SARS-CoV-2-induced mitochondrial membrane depolarization in Vero and Huh cells, as assessed by JC-1 staining [52]. Furthermore, evidence suggests that SARS-CoV-2 can compromise mitochondrial function in brain cells, potentially contributing to the neurological manifestations of COVID-19 [53]. The observed isoform switching in mitochondrial genes during infection may, therefore, provide mechanistic insights into the gene expression changes underlying the mitochondrial dysfunction observed during SARS-CoV-2 infection.

We investigated the potential overlap between differentially expressed transcripts and those exhibiting differential RNA modification patterns in response to infection. Surprisingly, we observed no intersection between these two sets of transcripts, despite the well-established role of RNA modifications in gene regulation [54]. This finding suggests that these two regulatory layers may operate independently during the host response to infection. The modifications we identified might regulate aspects of RNA metabolism beyond transcript levels, such as mRNA stability, nuclear export or translation efficiency, without directly affecting RNA abundance.

A previous study using VeroE6-H10 cells identified 1,474 proteins of interest through proteomic analysis, focusing on targets exhibiting significant changes in both protein expression and phosphorylation patterns following SARS-CoV-2 infection [3]. We compared our results dataset against these previously identified proteins. Our analysis revealed three instances of isoform switching that overlapped with the original dataset: HSPB1 (UniProt: P04792, Ensembl: ENSG00000106211), LAP3 (UniProt: P28838, Ensembl: ENSG00000002549) and CPOX (UniProt: P36551, Ensembl: ENSG00000080819). Moreover, we identified several differentially expressed genes that overlapped with the previous study’s proteins of interest list [3]. Among the downregulated genes during infection, we found ten overlapping proteins: HSPB1 (UniProt: P04792, Ensembl: ENSG00000106211), PSAP (UniProt: P07602, Ensembl: ENSG00000197746), PTPRF (UniProt: P10586, Ensembl: ENSG00000142949), CKMT1A (UniProt: P12532, Ensembl: ENSG00000223572), SERPINF1 (UniProt: P36955, Ensembl: ENSG00000132386), CSNK1D (UniProt: P48730, Ensembl: ENSG00000141551), PLK1 (UniProt: P53350, Ensembl: ENSG00000166851), RAD23A (UniProt: P54725, Ensembl: ENSG00000179262), ACTB (UniProt: P60709, Ensembl: ENSG00000075624) and FSCN1 (UniProt: Q16658, Ensembl: ENSG00000075618). Additionally, three proteins were found to be upregulated: CSTB (UniProt: P04080, Ensembl: ENSG00000160213), TPT1 (UniProt: P13693, Ensembl: ENSG00000133112) and GREB1 (UniProt: Q12849, Ensembl: ENSG00000132463). The validation of these targets through both proteomics and direct RNA sequencing approaches suggests they may serve as promising candidates for future mechanistic studies investigating host–pathogen interactions during SARS-CoV-2 infection.

## Supplementary material

10.1099/acmi.0.001019.v3 Uncited Supplementary Material 1.

10.1099/acmi.0.001019.v3 Uncited Supplementary Material 2.

## Abbreviations

ATSS: alternative transcription start site
ATTS: alternative transcription termination site
COVID-19: coronavirus disease 2019
DTT: dithiothreitol
GO: gene ontology
gRNA: genomic RNA
h.p.i.: hours post-infection
KEGG: Kyoto Encyclopedia of Genes and Genomes
m6A: N6-methyladenosine
*N*: nucleocapsid gene
nc-sgRNAs: non-canonical sgRNAs
NEAA: non‐essential amino acids
ONT: Oxford Nanopore Technologies
*S*: spike gene
SARS-CoV-2: severe acute respiratory syndrome coronavirus 2
sgRNAs: subgenomic RNAs
