# Supplementary material for: Direct RNA sequencing reveals m6A modifications and isoform changes in SARS-CoV-2-infected HEK cells
Source: Access Microbiol. 2025 Sep 17;7(9):001019.v3. doi: 10.1099/acmi.0.001019.v3 (PMC12451313; doi:10.1099/acmi.0.001019.v3)
Supplement: Uncited Supplementary Material 1. [file acmi-7-01019-s001.pdf]

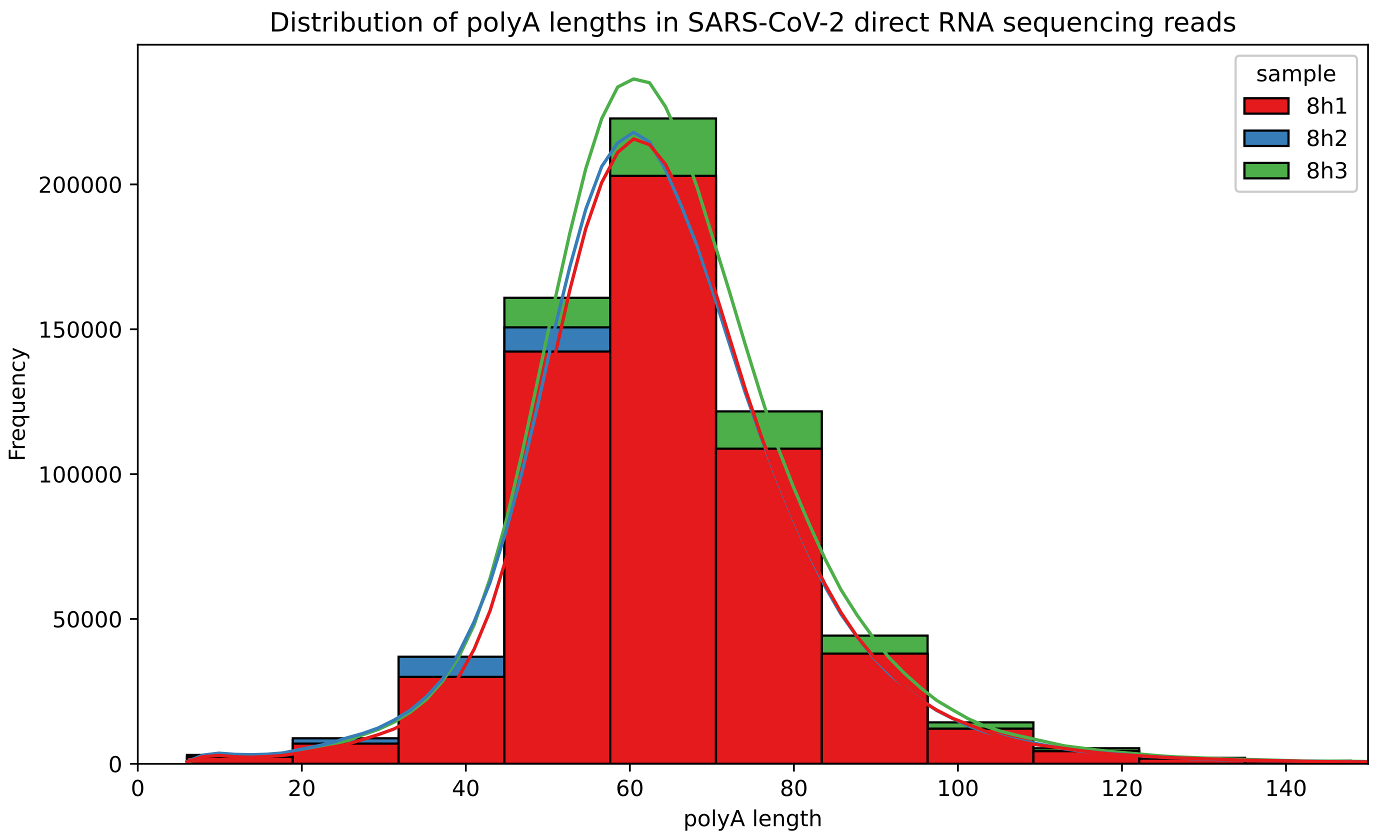

Supplementary Figure S1. Distribution of polyA lengths in SARS-CoV-2 direct RNA sequencing reads. The figure shows the distribution of polyA lengths for three different samples (8h1, 8h2, and 8h3) obtained from SARS-CoV-2 direct RNA sequencing reads (SARS-CoV-2 mapped reads). The histogram displays the frequency of polyA lengths, with each sample represented by a different color. The x-axis represents the polyA length, ranging from 0 to 150 nucleotides, and the y-axis represents the frequency of occurrences.

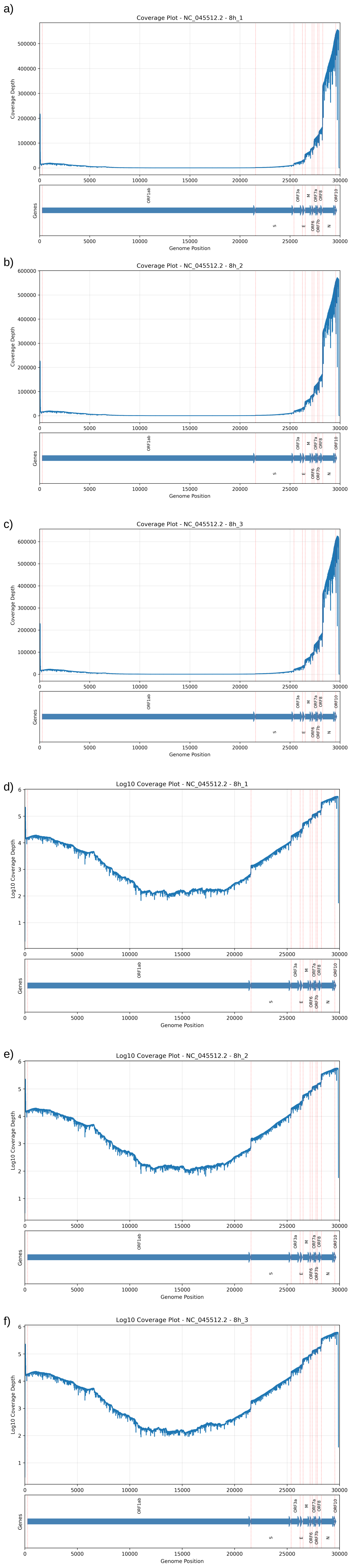

Supplementary Figure 2. Genome coverage depth and gene annotations for SARS-CoV-2 in samples a) 8h1, b) 8h2, and c) 8h3. The upper panels show the sequencing coverage depth (blue markers) across the genome, with red dotted lines indicating gene start positions. The lower panels depict gene architecture, with arrows representing gene positions and transcriptional directions. Log10-transformed coverage depth plots are shown in d) 8h1, e) 8h2, and f) 8h3.

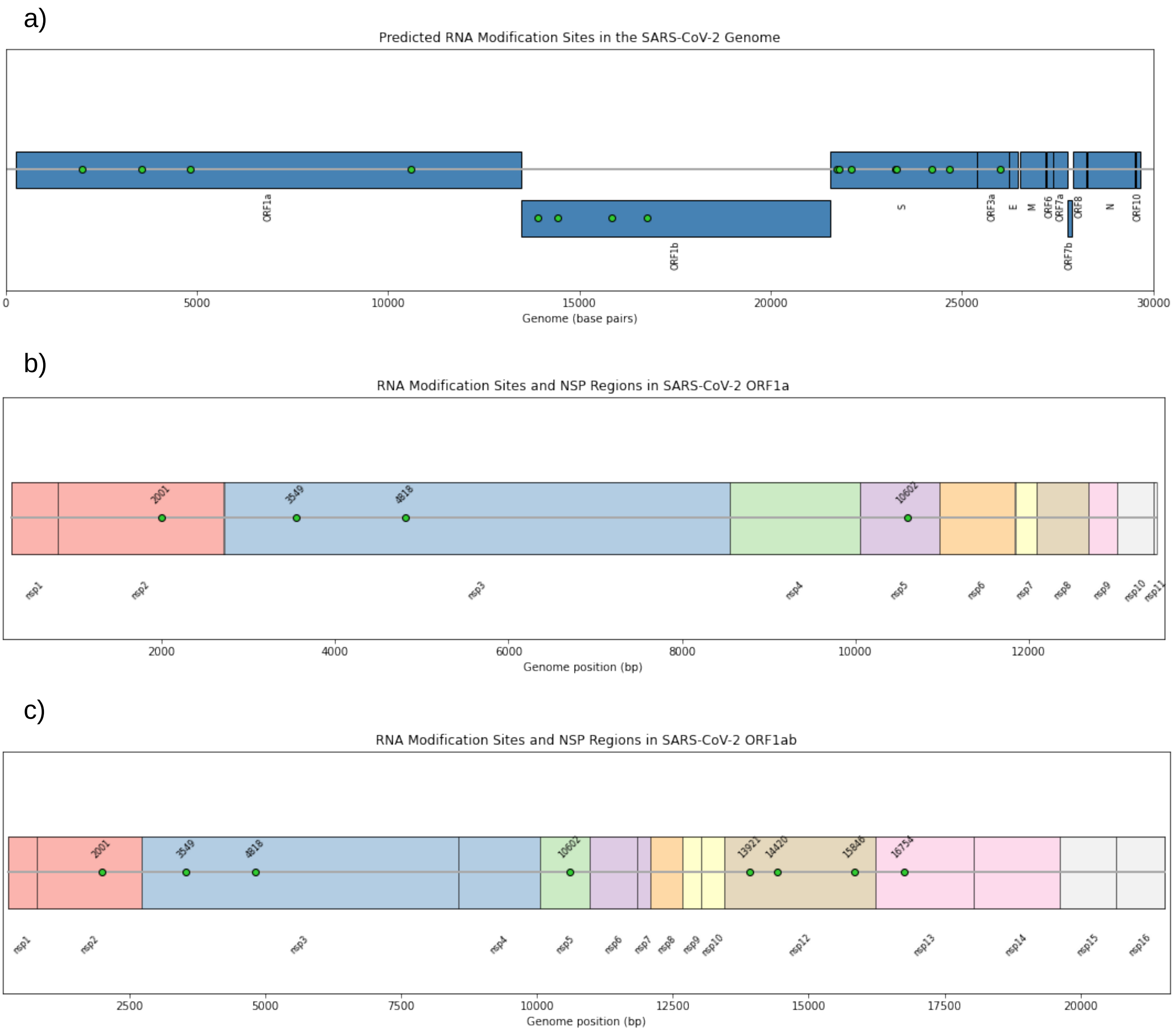

Supplementary Figure S3. a) Genome map of SARS-CoV-2 highlighting predicted RNA modification sites. The genome is represented as a grey line, with genes shown as blue boxes. Base pair positions with predicted RNA modifications are marked with green dots. The x-axis represents the genome length in base pairs, and gene names are labeled along the genome.
b) Zoomed-in view of the ORF1a region of the SARS-CoV-2 genome. Non-structural proteins (NSPs) within ORF1a are shown as distinct colored boxes to enhance clarity. Predicted RNA modification sites are indicated with green dots. The x-axis represents the gene length in base pairs, and protein names are labeled along the region.
c) Zoomed-in view of the ORF1ab region of the SARS-CoV-2 genome. NSPs within ORF1ab are shown as distinct colored boxes to differentiate them. Predicted RNA modification sites are marked with green dots. The x-axis represents the gene length in base pairs, and protein names are labeled along the region.

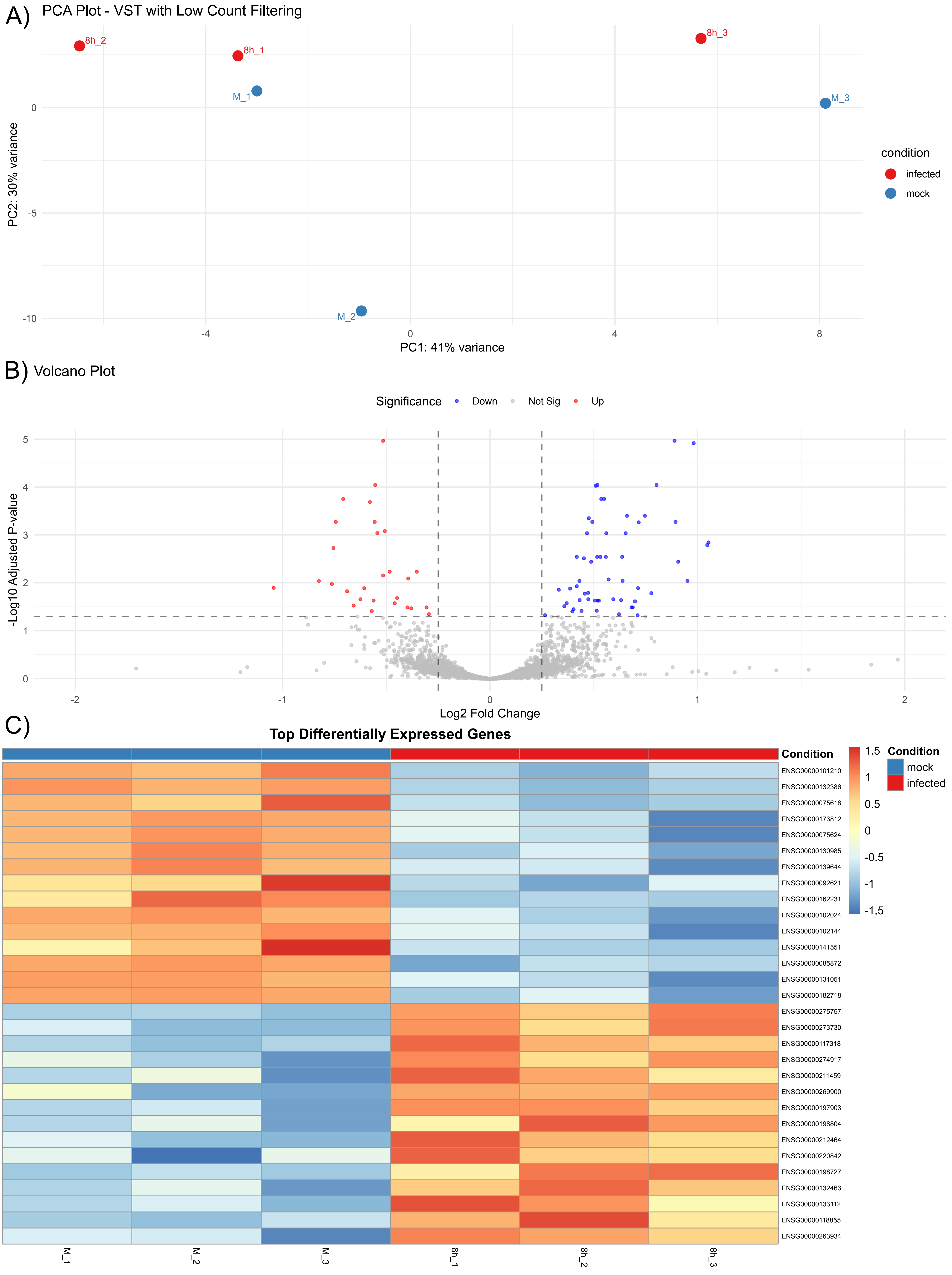

Supplementary Figure S4. Differential gene expression analysis between infected and mock samples.
A) Principal component analysis (PCA) of variance-stabilized transformed gene expression data. Red dots represent infected samples and blue dots represent mock samples. B) Volcano plot displaying differential gene expression with $\log_2$ fold change (x-axis) versus $-\log_{10}$ adjusted p-value (y-axis). Blue dots indicate genes significantly downregulated in infected samples, red dots show genes significantly upregulated in infected samples. Dashed lines represent significance thresholds (adjusted p-value < 0.05 and $|\log_2$ fold change$| > 0.25$). C) Heatmap of the top 30 differentially expressed genes (15 most significantly upregulated and 15 most significantly downregulated, ranked by adjusted p-value). Colors represent z-score normalized expression levels across samples, with sample annotations indicating experimental conditions (Mock 1,2,3 and 8h infected 1,2,3).

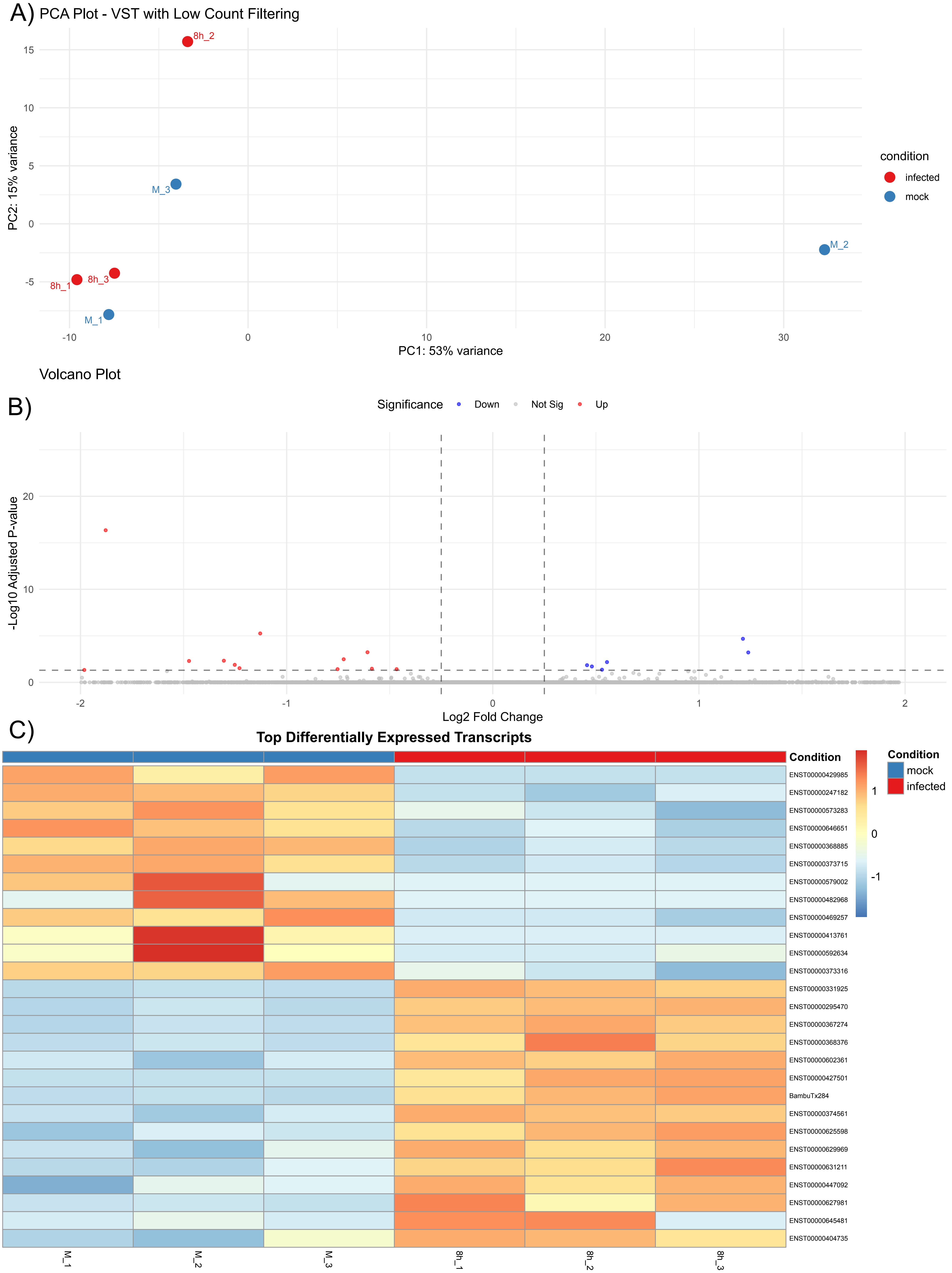

Supplementary Figure S5. Differential transcript expression analysis between infected and mock samples.
A) Principal component analysis (PCA) of variance-stabilized transformed transcript expression data. Red dots represent infected samples and blue dots represent mock samples. B) Volcano plot displaying differential transcript expression with $\log_2$ fold change (x-axis) versus $-\log_{10}$ adjusted p-value (y-axis). Blue dots indicate transcripts significantly downregulated in infected samples, red dots show transcripts significantly upregulated in infected samples. Dashed lines represent significance thresholds (adjusted p-value < 0.05 and $|\log_2$ fold change$| > 0.25$). C) Heatmap of the top 27 differentially expressed transcripts (15 most significantly upregulated and 12 most significantly downregulated, ranked by adjusted p-value). Colors represent z-score normalized expression levels across samples, with sample annotations indicating experimental conditions (Mock 1,2,3 and 8h infected 1,2,3).

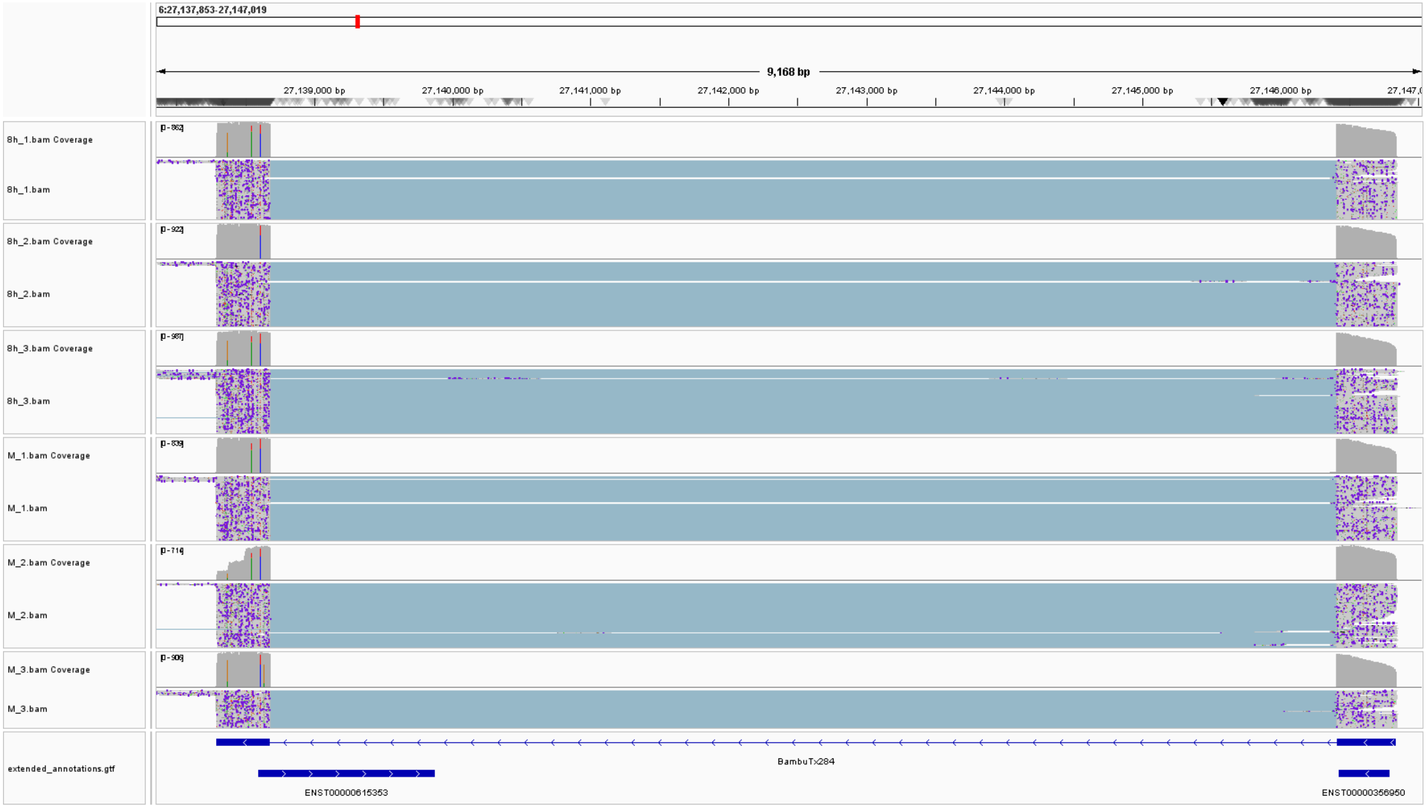

Supplementary Figure S6: Identification of a Novel Transcript BambuTx284 encoded by the gene ENSG00000197903 with an extended non-coding region. The IGV browser view shows RNA-seq coverage across the genomic region of ENSG00000197903, with multiple samples aligned. The annotated reference transcript ENST00000356950 (the only annotated reference transcript of this gene) and the novel isoform BambuTx284 are shown. The RNA-seq data (represented in blue and grey tracks) confirm the presence of an extended region beyond the annotated transcript ENST00000356950. This extension is supported by high coverage of long-read direct RNA sequencing, suggesting a novel isoform with the same protein-coding region but a longer 3' untranslated region (UTR). Data are shown for both mock (M) and infected (8h) samples, highlighting consistent read support for the novel transcript across conditions.

The isoform switch in ENSG00000002549 gene (mock vs infected)

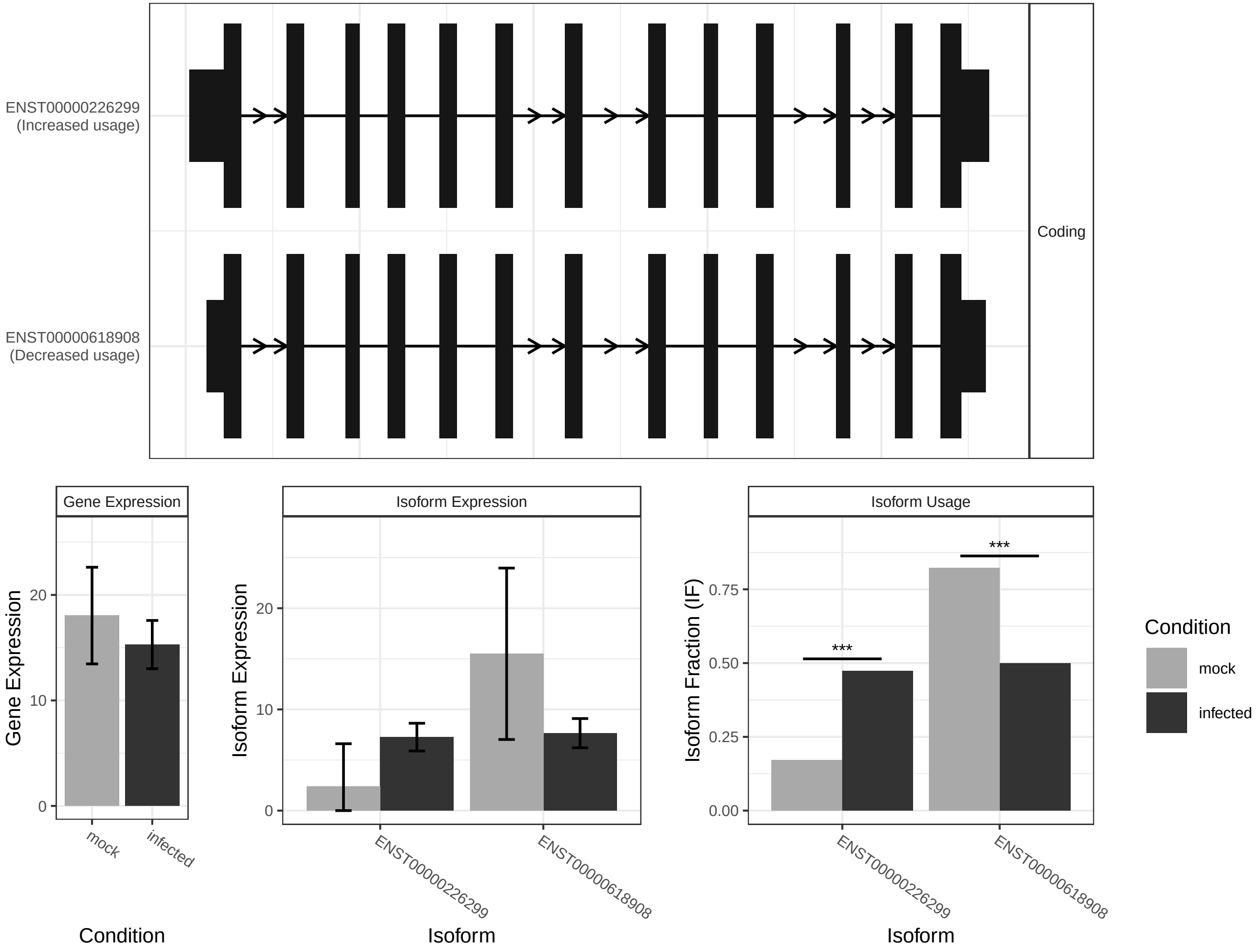

The isoform switch in ENSG00000054598 gene (mock vs infected)

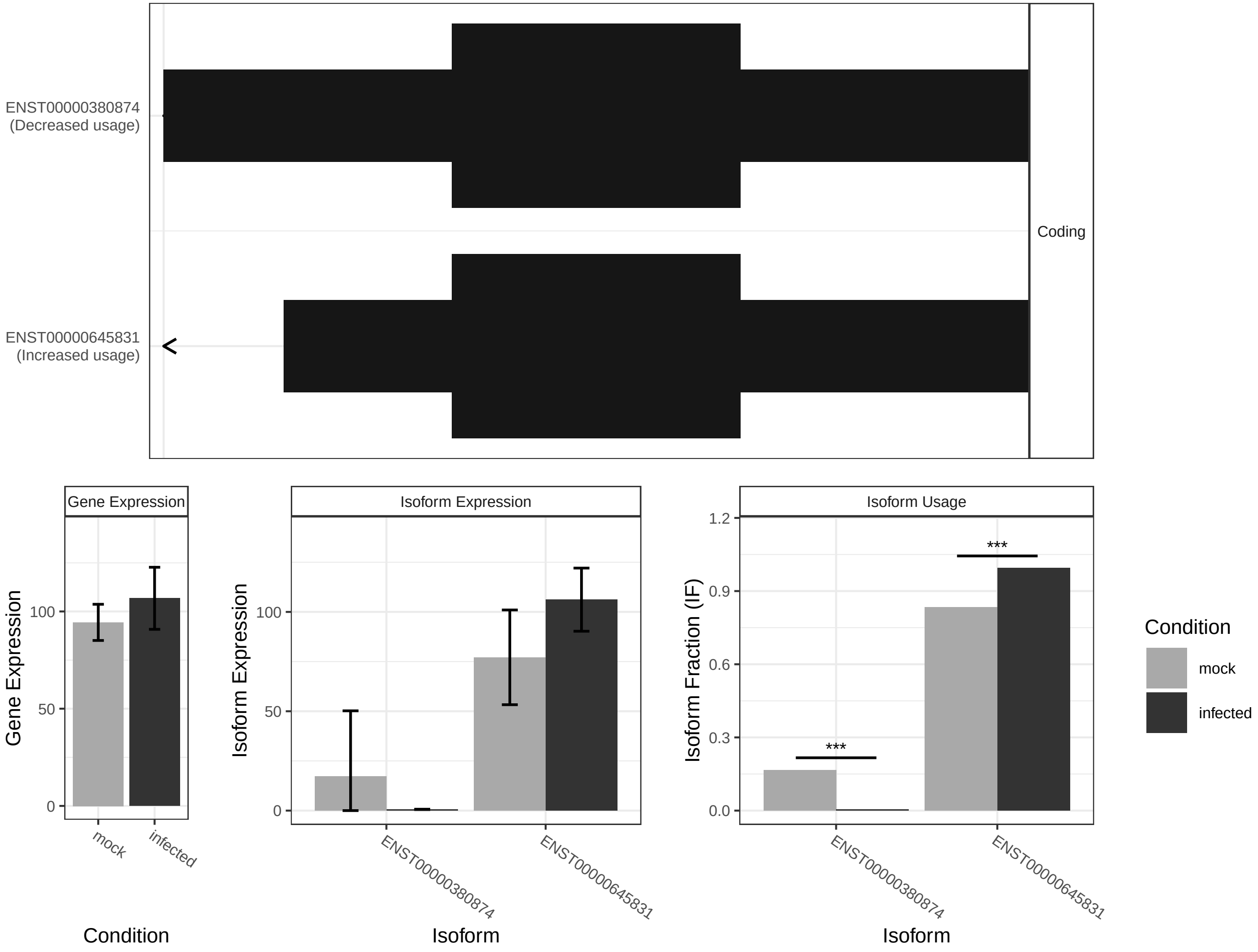

The isoform switch in ENSG00000077152 gene (mock vs infected)

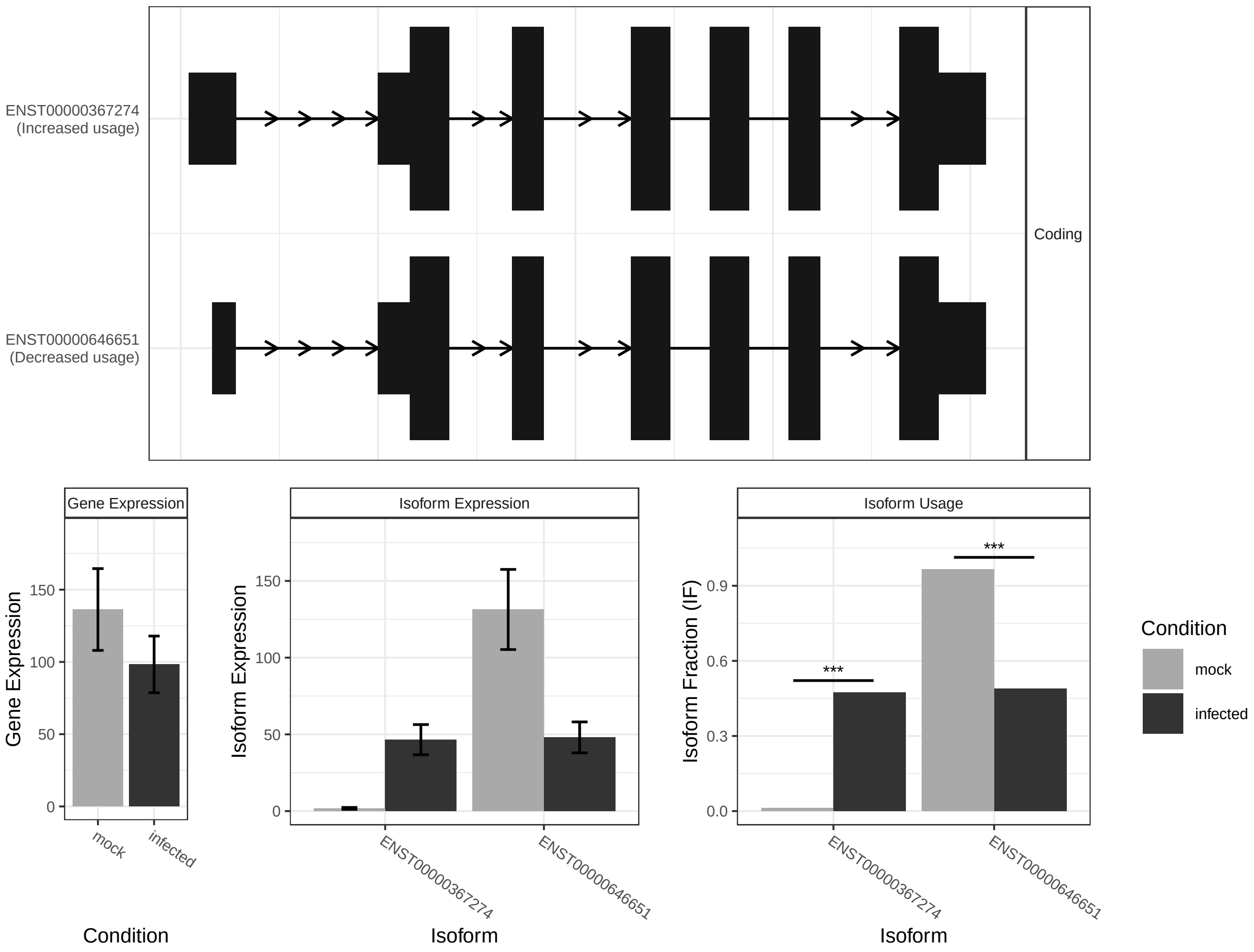

The isoform switch in ENSG00000080819 gene (mock vs infected)

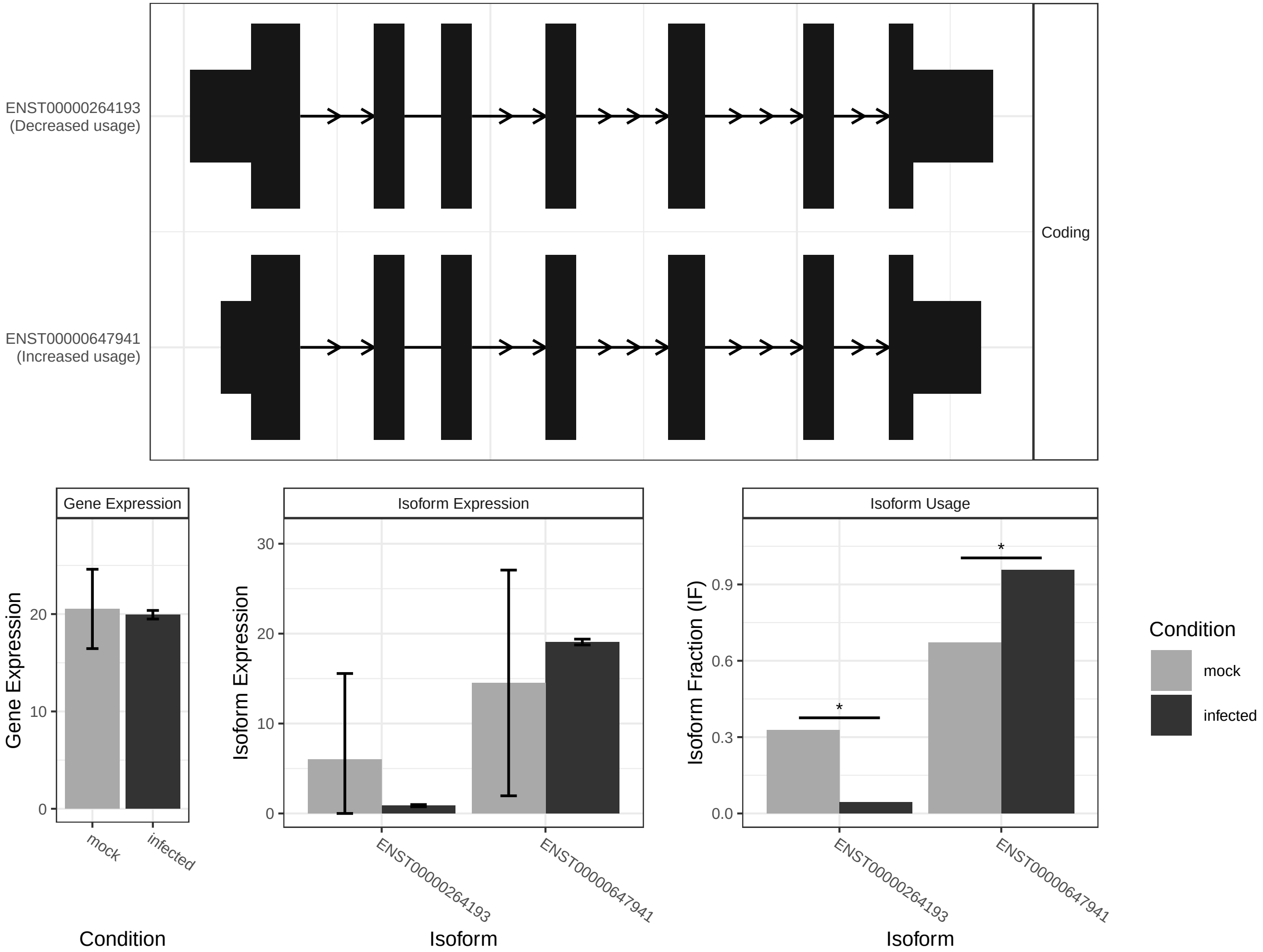

The isoform switch in ENSG00000081692 gene (mock vs infected)

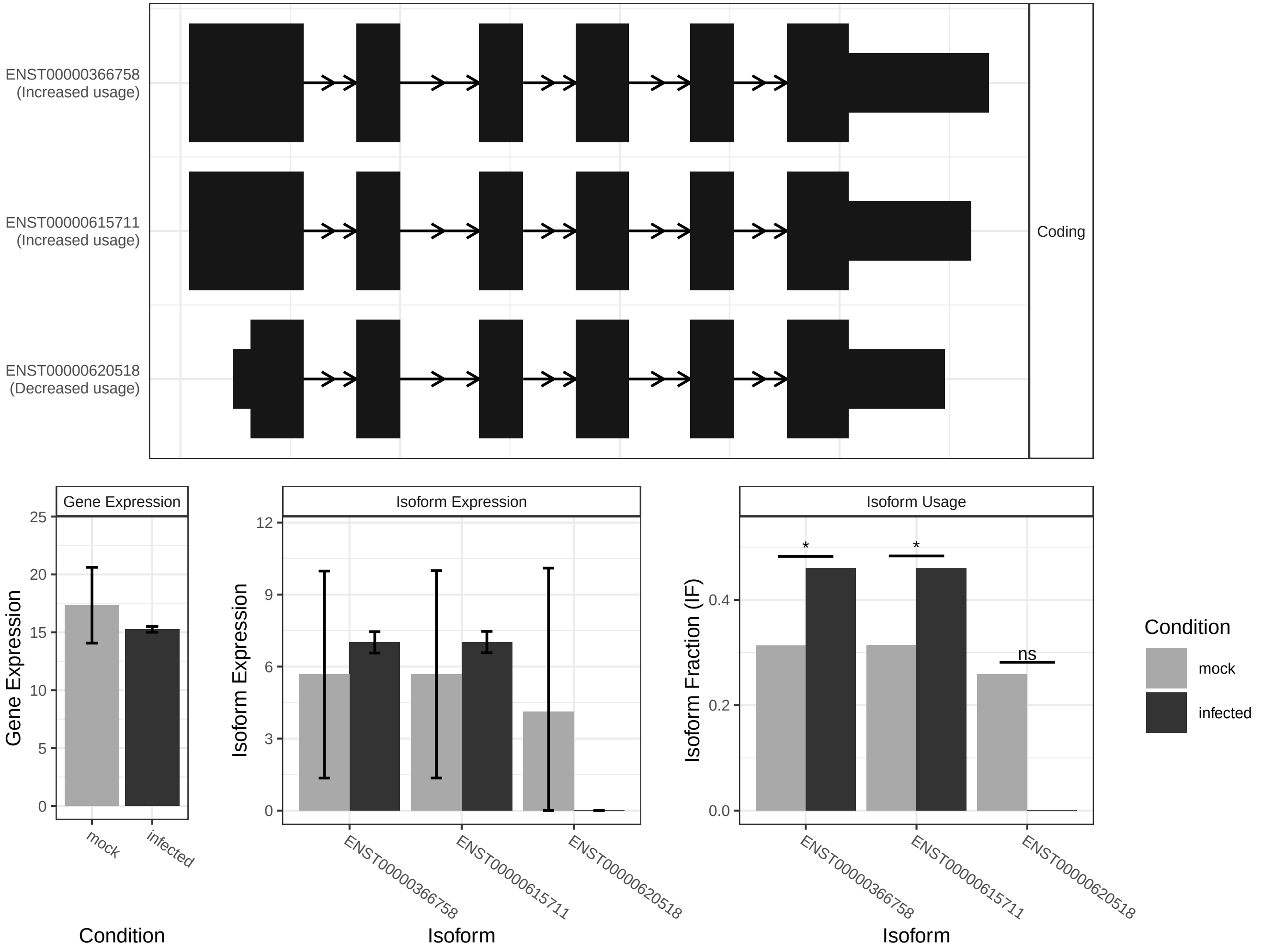

The isoform switch in ENSG00000087460 gene (mock vs infected)

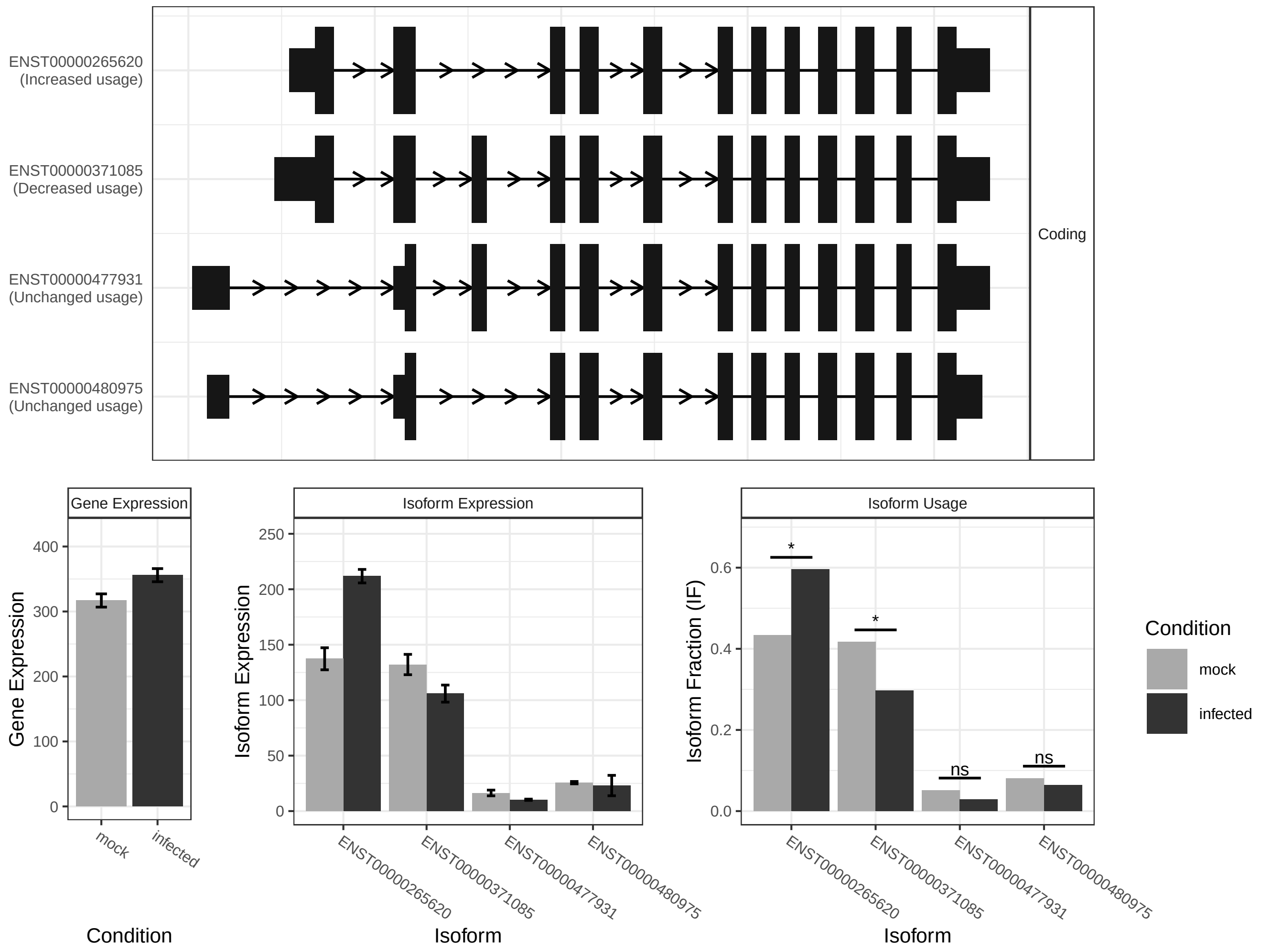

The isoform switch in ENSG00000106211 gene (mock vs infected)

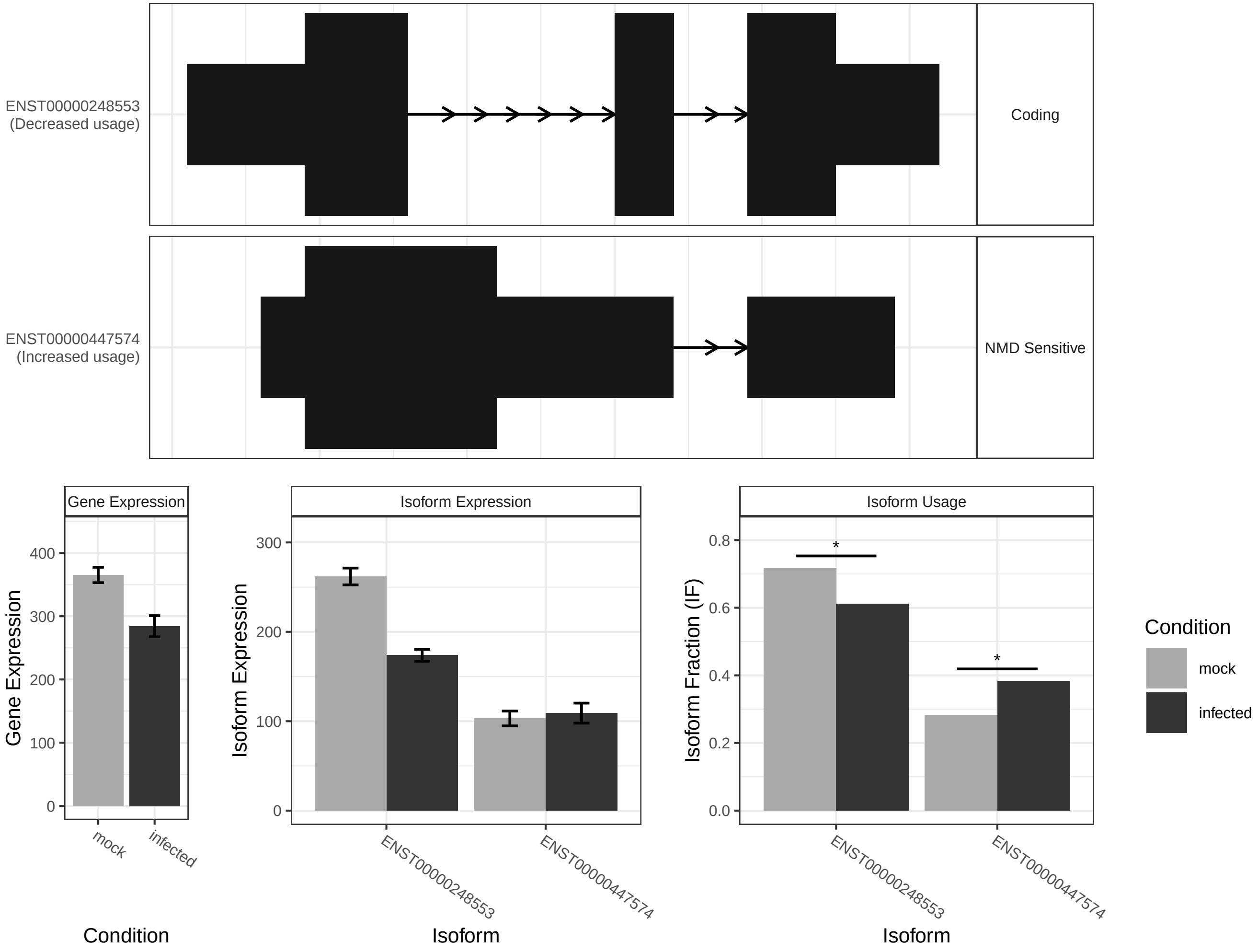

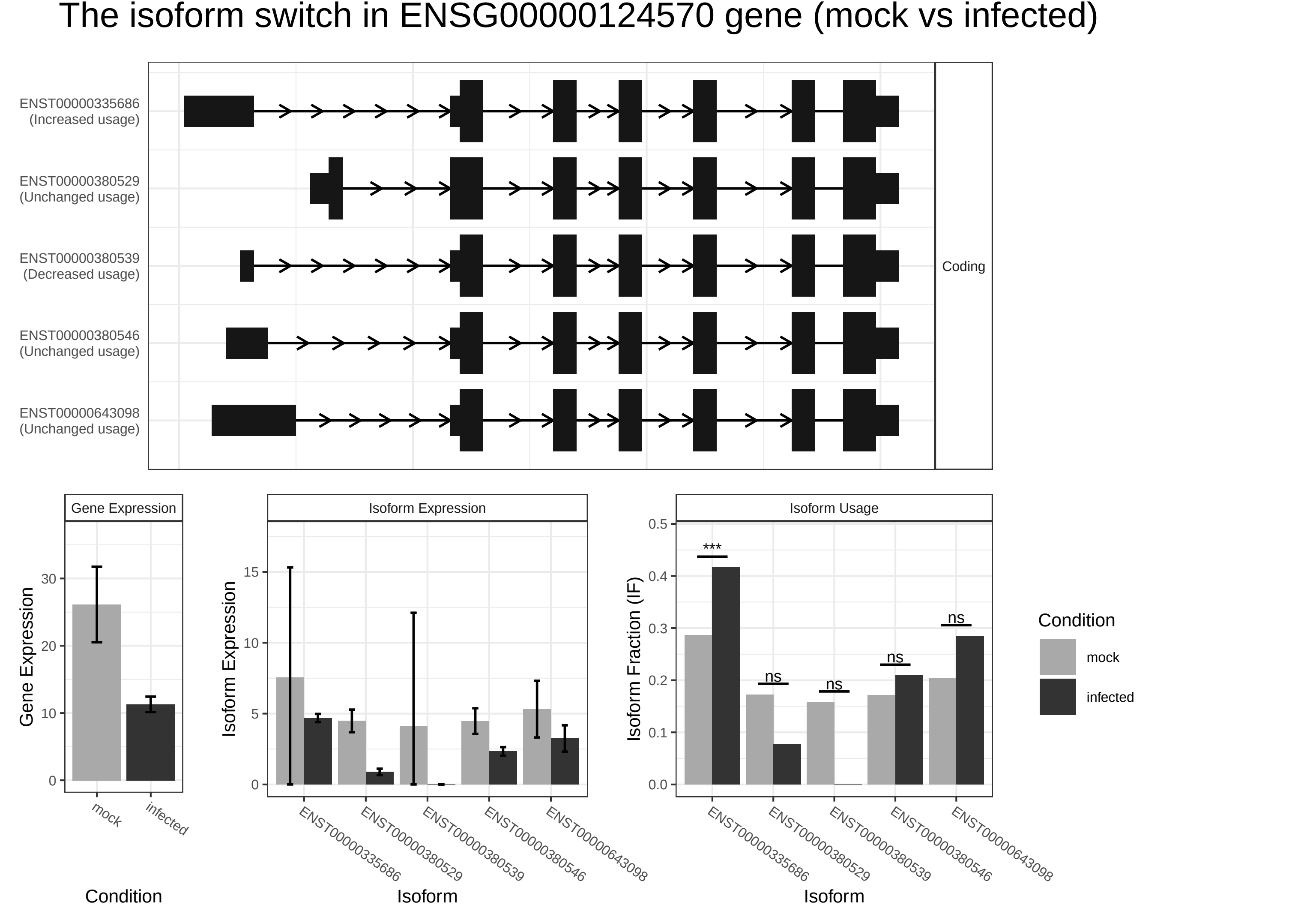
The isoform switch in ENSG00000124570 gene (mock vs infected)
ENST00000335686 (Increased usage)
ENST00000380529 (Unchanged usage)
ENST00000380539 (Decreased usage)
ENST00000380546 (Unchanged usage)
ENST00000643098 (Unchanged usage)
Coding
Gene Expression
Isoform Expression
Isoform Usage
Condition
mock
infected
Isoform
Isoform Fraction (IF)
***
ns

The isoform switch in ENSG00000126267 gene (mock vs infected)

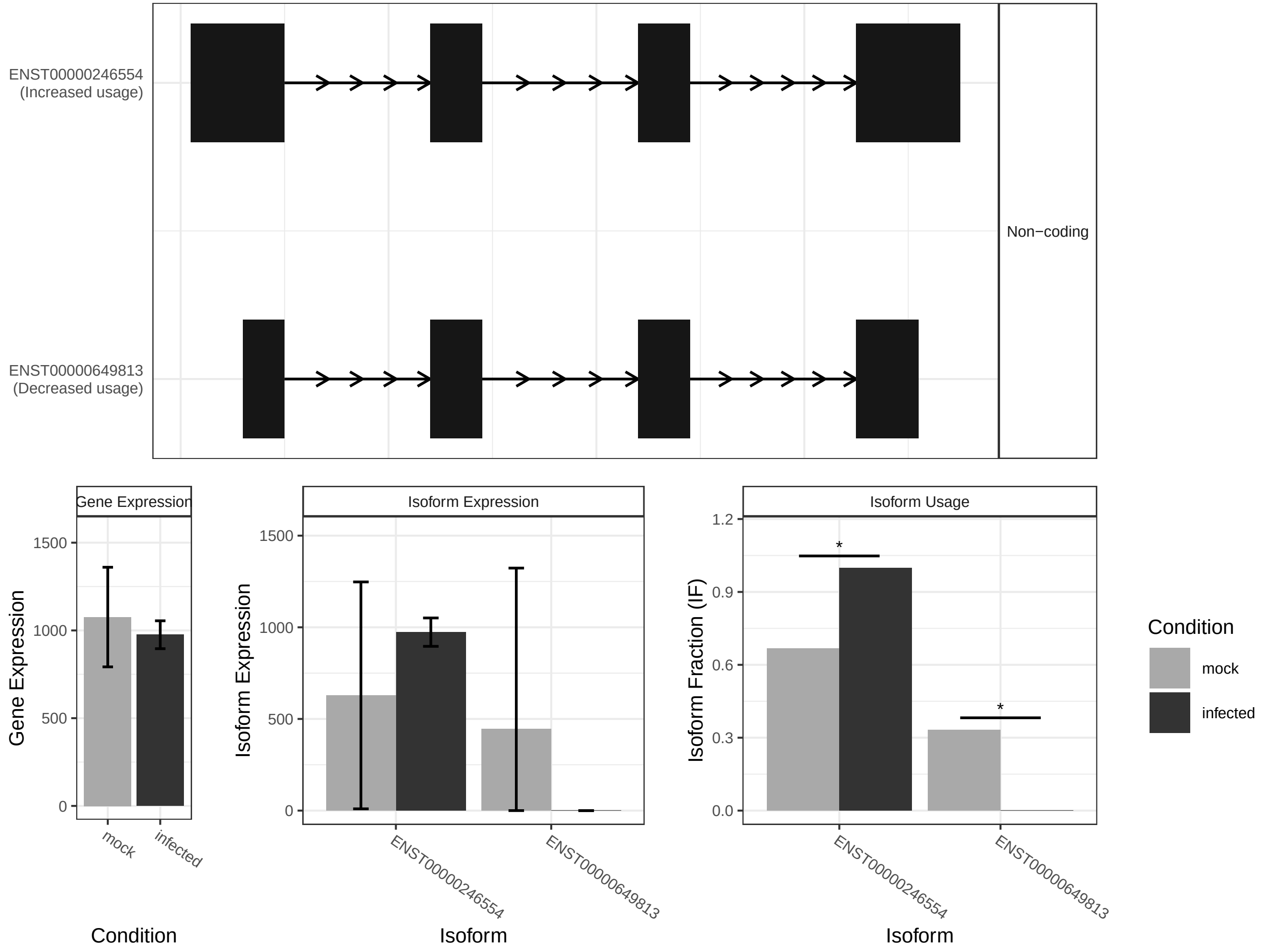

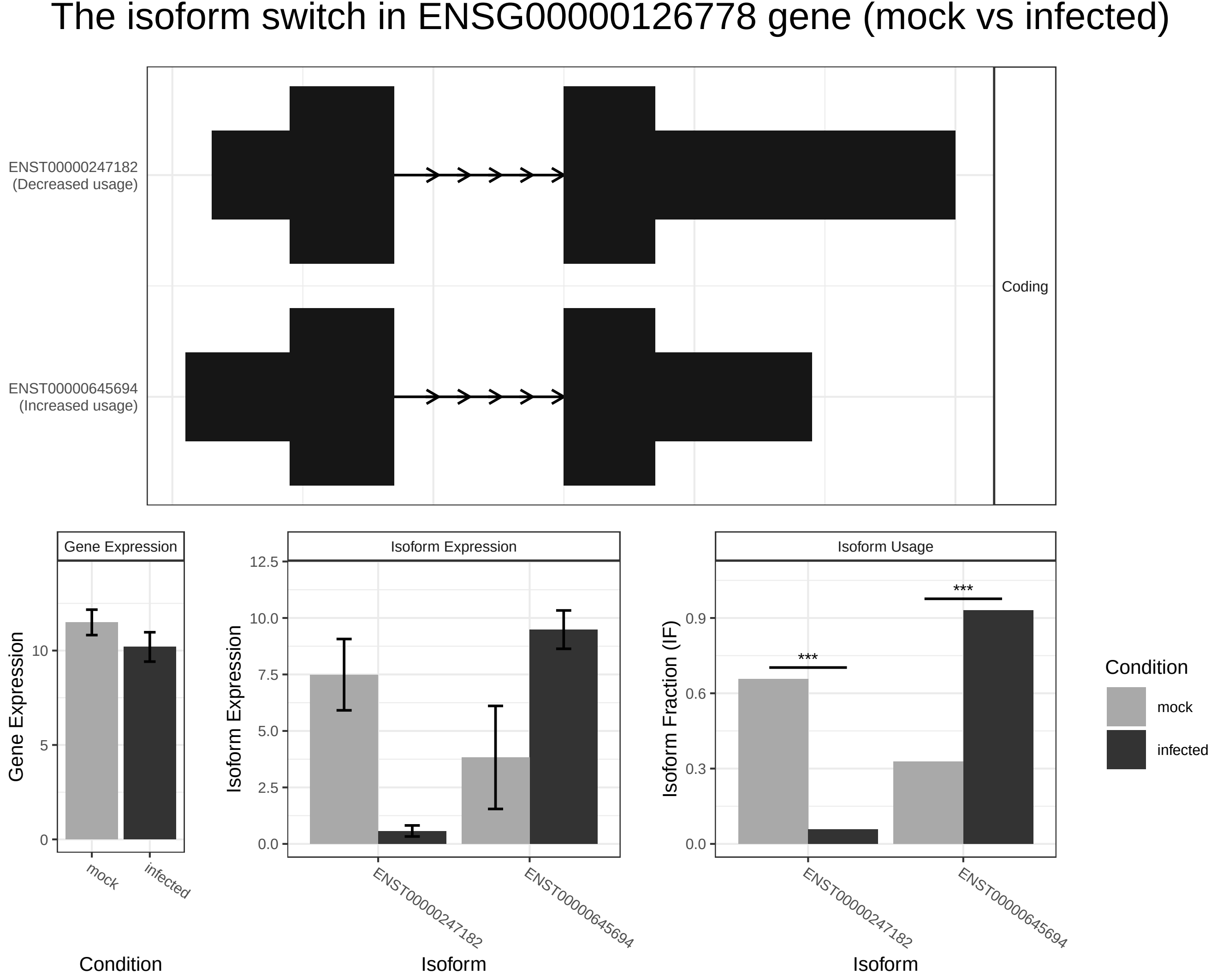
The isoform switch in ENSG00000126778 gene (mock vs infected)
ENST00000247182
(Decreased usage)
ENST00000645694
(Increased usage)
Coding
Gene Expression
Isoform Expression
Isoform Usage
Gene Expression
Isoform Expression
Isoform Fraction (IF)
0
5
10
0.0
2.5
5.0
7.5
10.0
12.5
0.0
0.3
0.6
0.9
***
***
mock
infected
ENST00000247182
ENST00000645694
ENST00000247182
ENST00000645694
Condition
Isoform
Isoform
Condition
mock
infected

The isoform switch in ENSG00000134108 gene (mock vs infected)

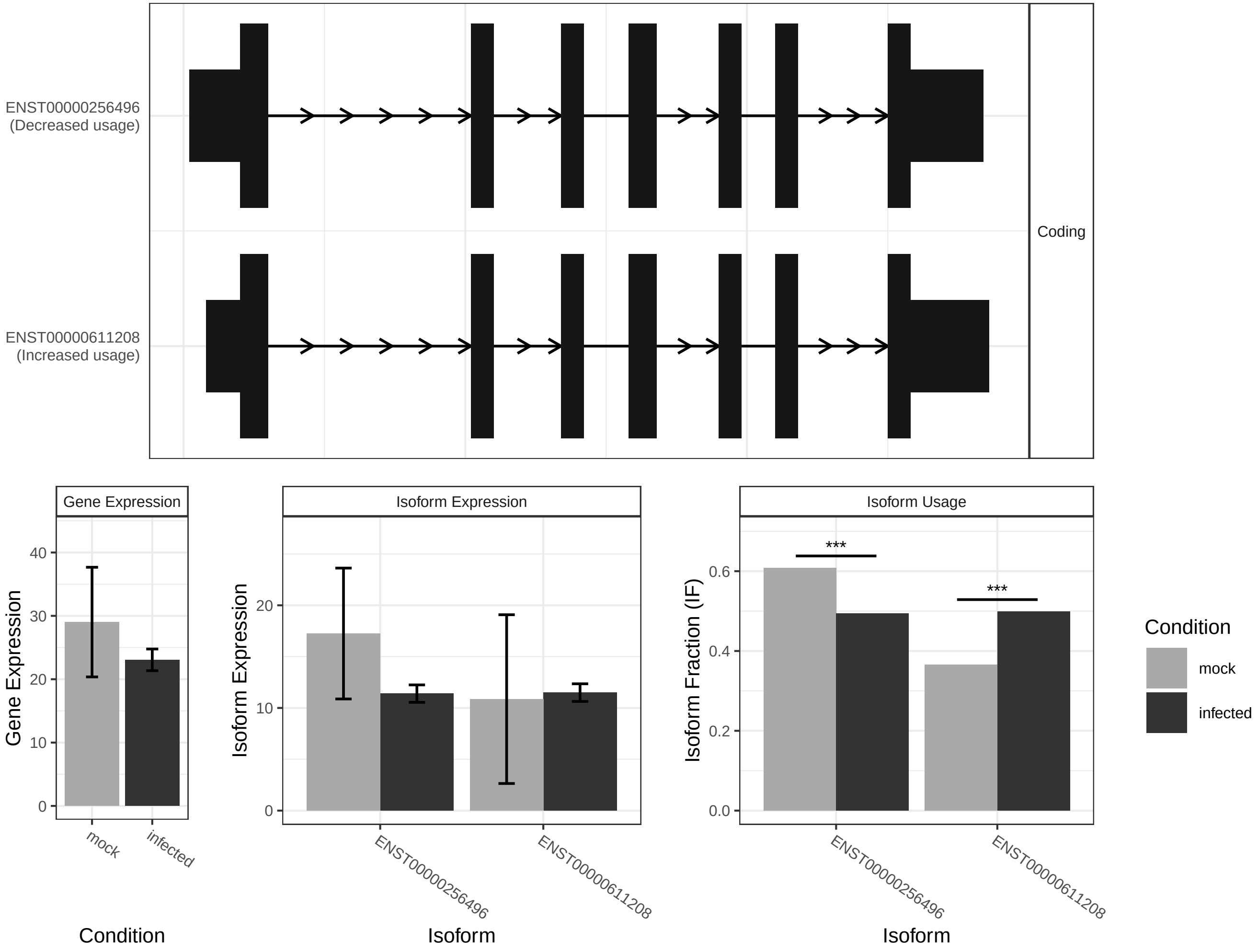

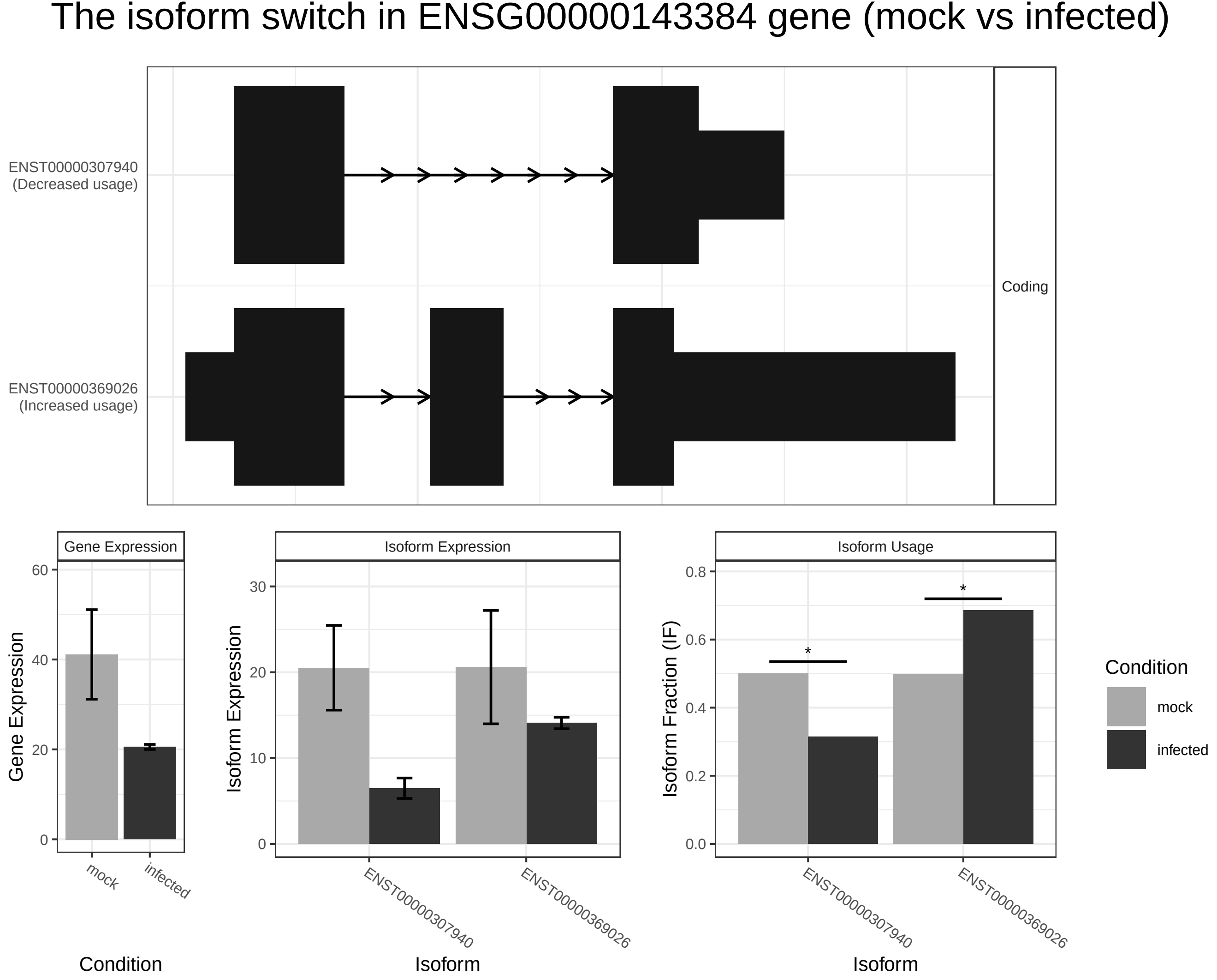
The isoform switch in ENSG00000143384 gene (mock vs infected)
ENST00000307940
(Decreased usage)
ENST00000369026
(Increased usage)
Coding
Gene Expression
Gene Expression
60
40
20
0
mock
infected
Condition
Isoform Expression
Isoform Expression
30
20
10
0
ENST00000307940
ENST00000369026
Isoform
Isoform Usage
Isoform Fraction (IF)
0.8
0.6
0.4
0.2
0.0
*
*
ENST00000307940
ENST00000369026
Isoform
Condition
mock
infected

The isoform switch in ENSG00000144659 gene (mock vs infected)

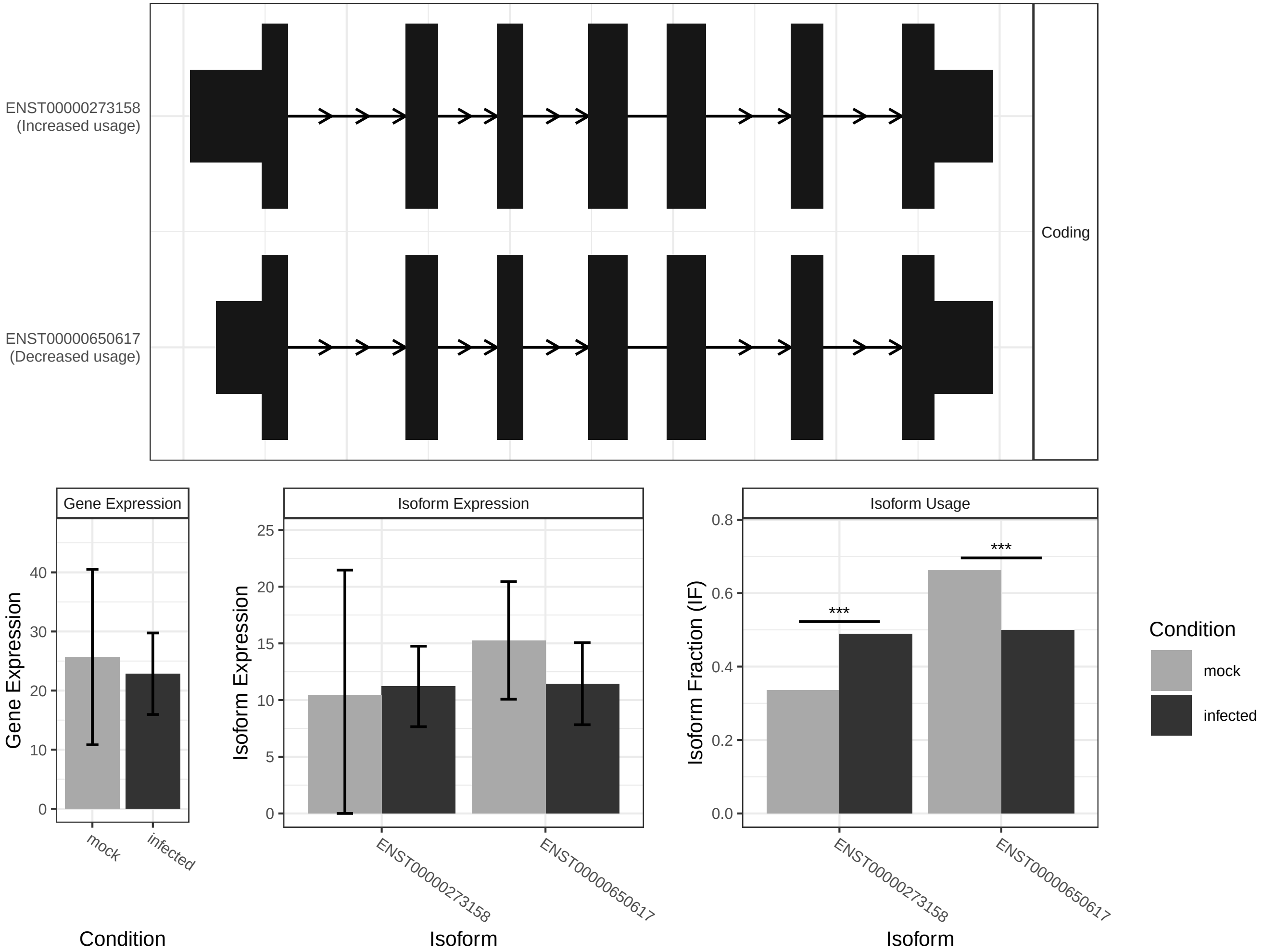

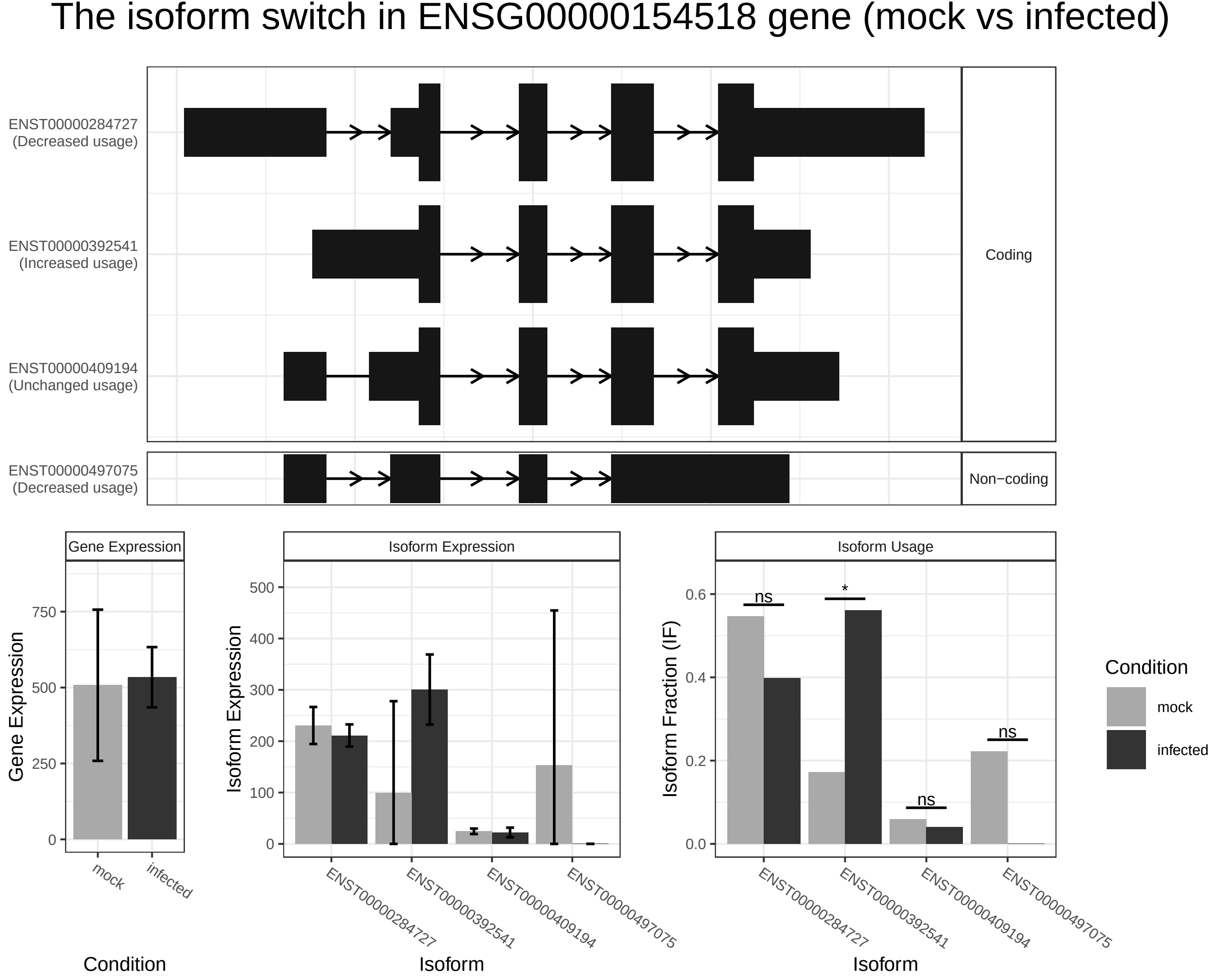
The isoform switch in ENSG00000154518 gene (mock vs infected)
ENST00000284727 (Decreased usage)
ENST00000392541 (Increased usage)
ENST00000409194 (Unchanged usage)
ENST00000497075 (Decreased usage)
Coding
Non−coding
Gene Expression
Isoform Expression
Isoform Usage
Condition
mock
infected
Isoform
Isoform Fraction (IF)
ns
*
ns
ns

The isoform switch in ENSG00000160932 gene (mock vs infected)

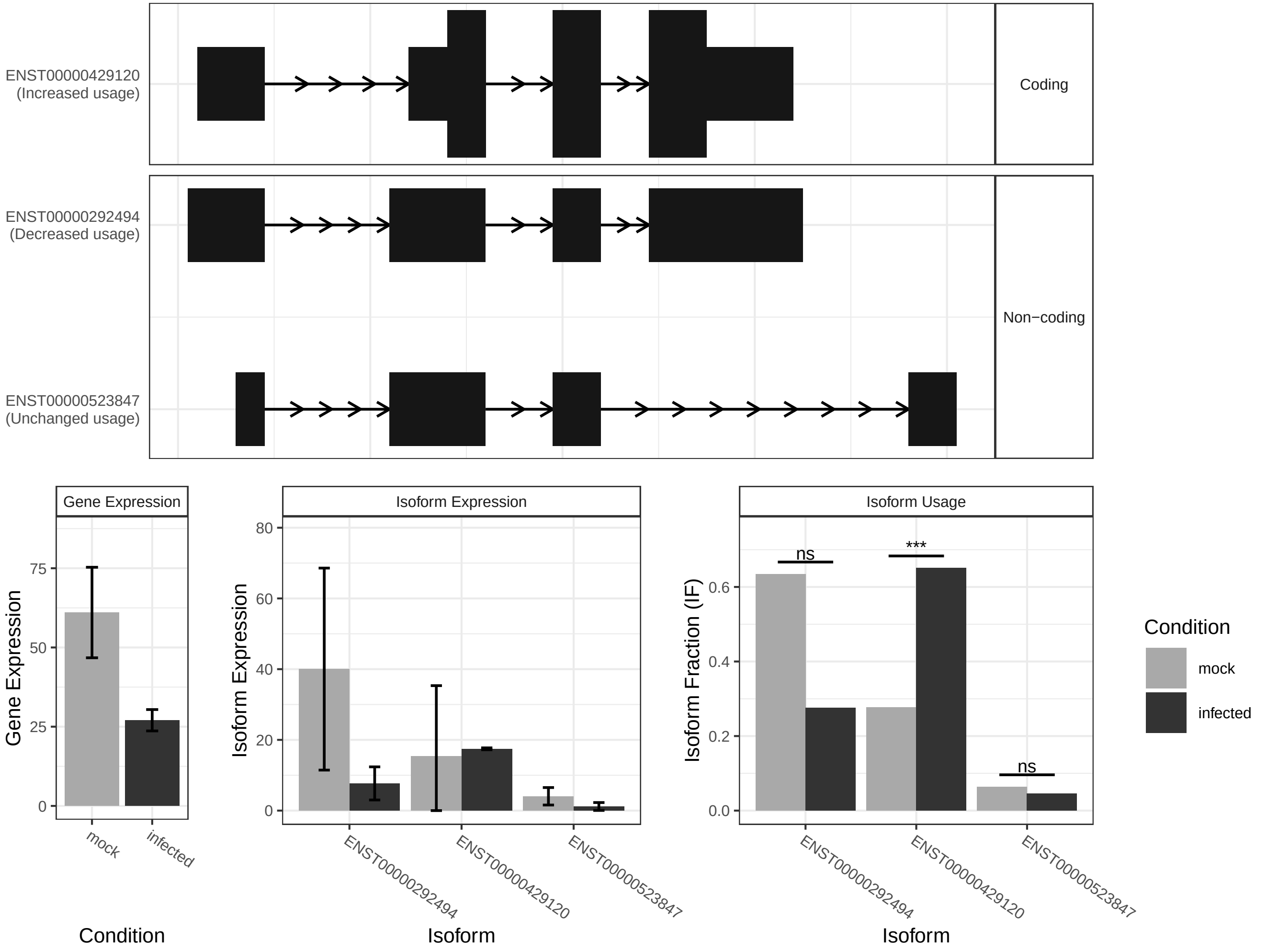

The isoform switch in ENSG00000162604 gene (mock vs infected)

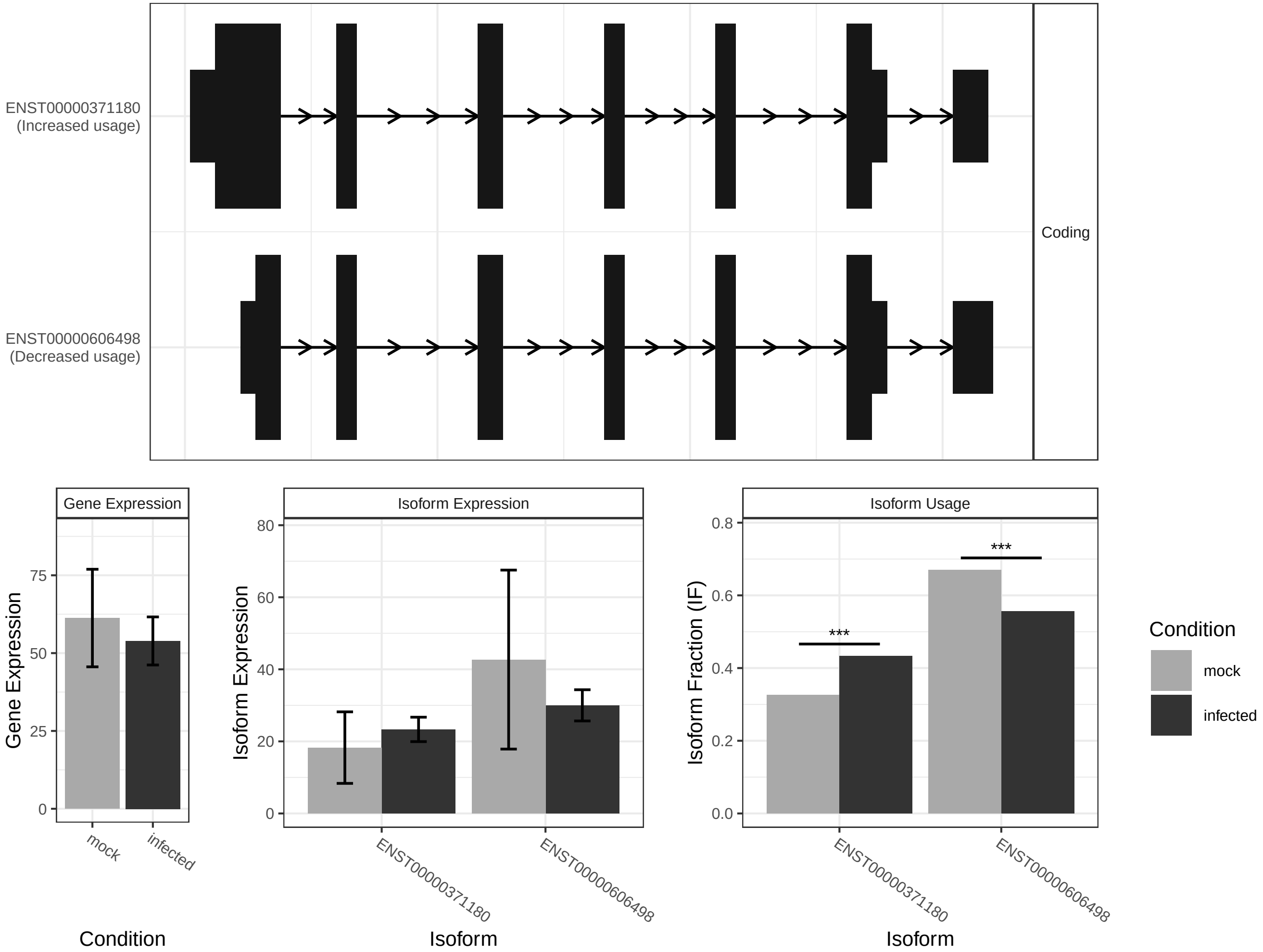

The isoform switch in ENSG00000162972 gene (mock vs infected)

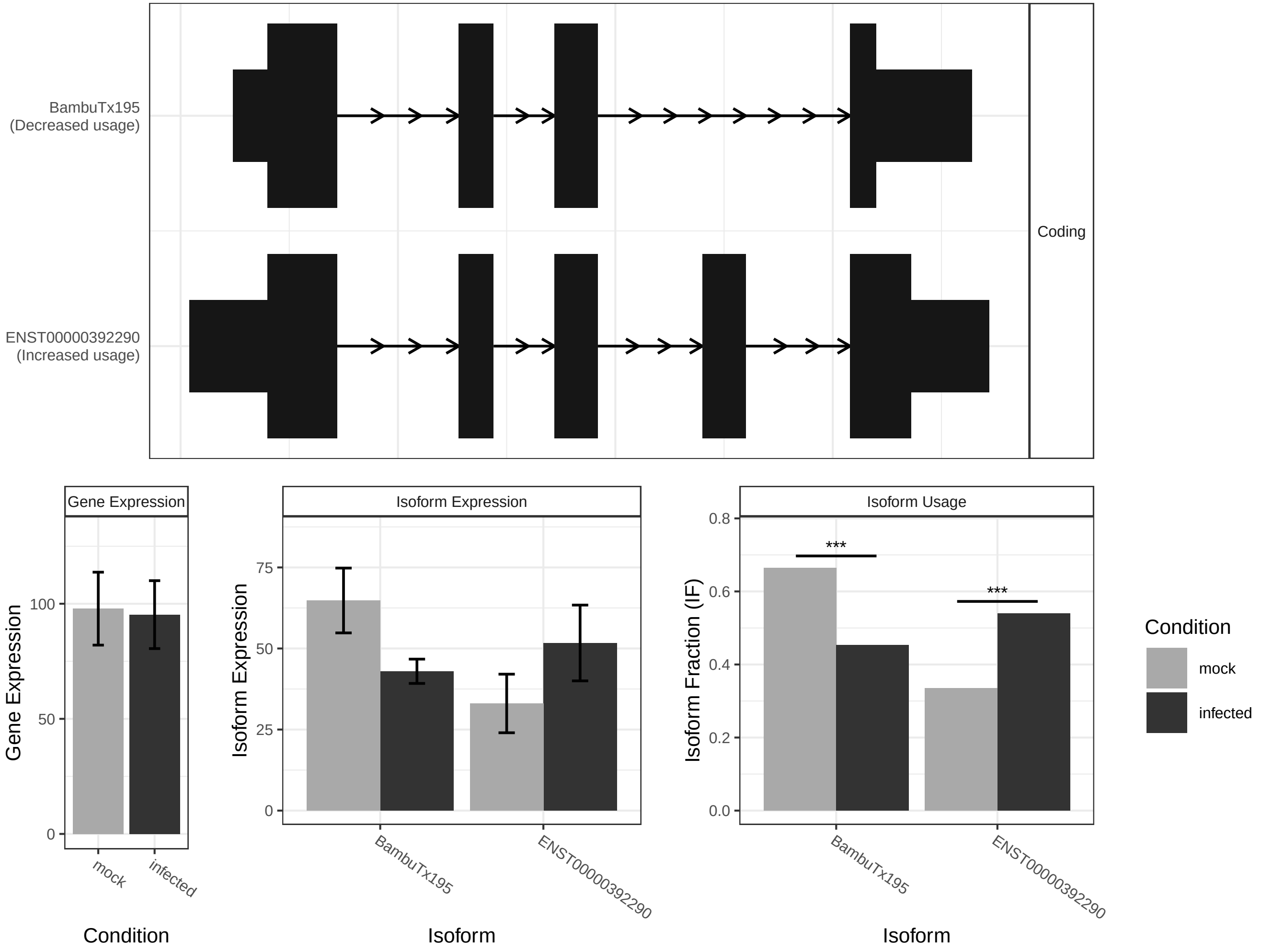

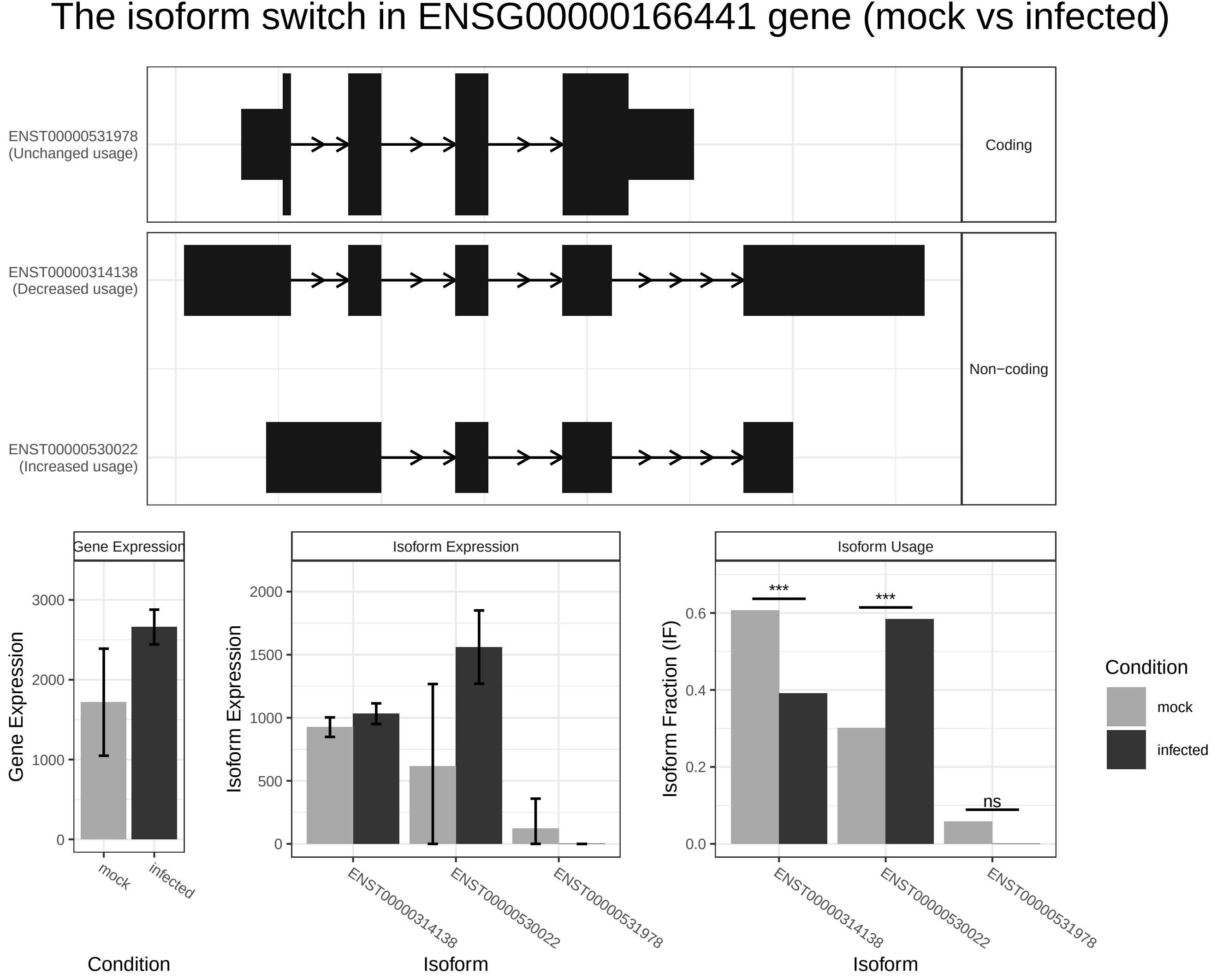
The isoform switch in ENSG00000166441 gene (mock vs infected)
ENST00000531978 (Unchanged usage)
ENST00000314138 (Decreased usage)
ENST00000530022 (Increased usage)
Coding
Non−coding
Gene Expression
Isoform Expression
Isoform Usage
Gene Expression
Isoform Expression
Isoform Fraction (IF)
0
1000
2000
3000
0
500
1000
1500
2000
0.0
0.2
0.4
0.6
***
***
ns
mock
infected
ENST00000314138
ENST00000530022
ENST00000531978
Condition
Isoform
Isoform
Condition
mock
infected

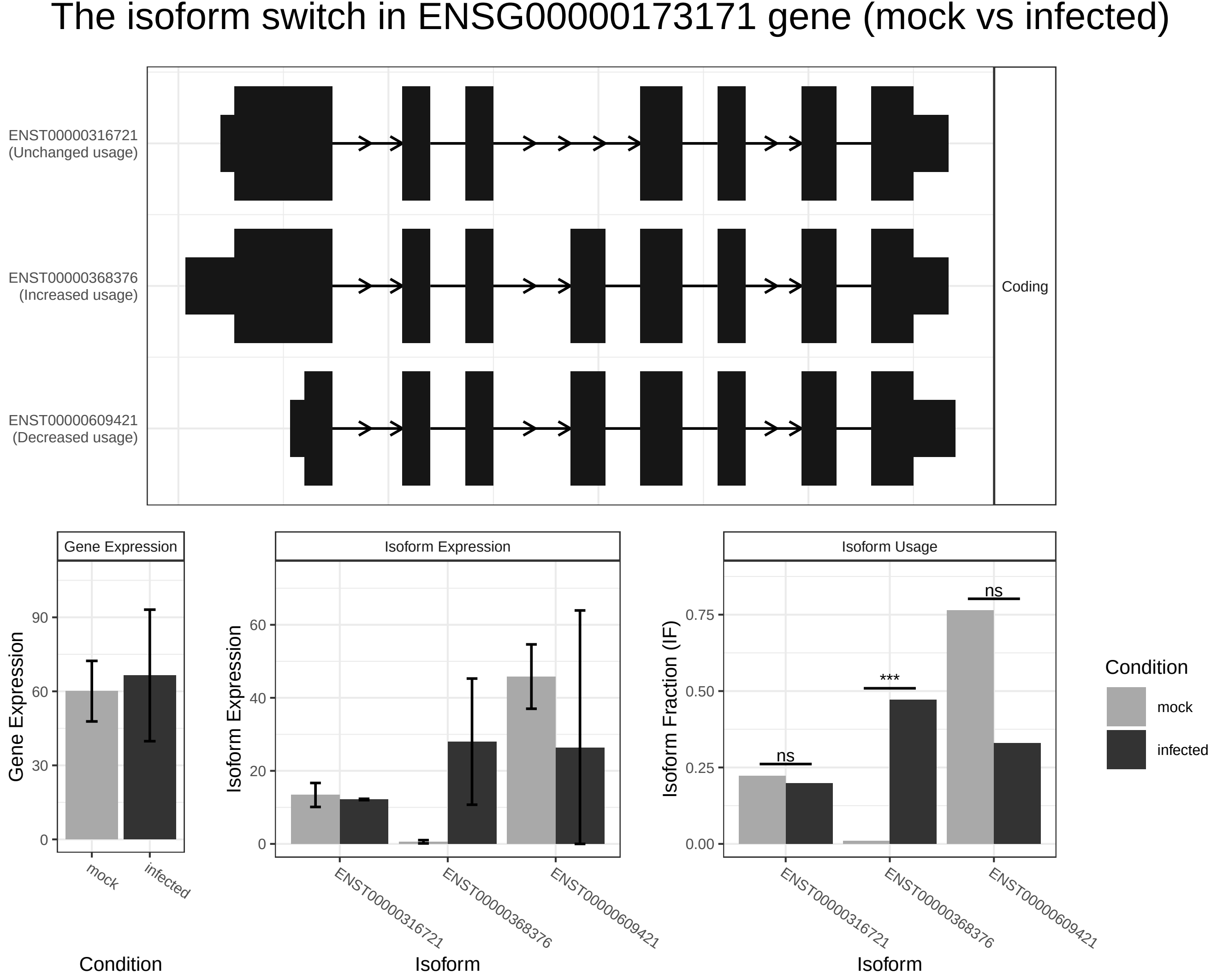

The isoform switch in ENSG00000173171 gene (mock vs infected)
ENST00000316721 (Unchanged usage)
ENST00000368376 (Increased usage)
ENST00000609421 (Decreased usage)
Coding
Gene Expression
Isoform Expression
Isoform Usage
Gene Expression
Isoform Expression
Isoform Fraction (IF)
Condition
Isoform
mock
infected
ns
***

The isoform switch in ENSG00000184009 gene (mock vs infected)

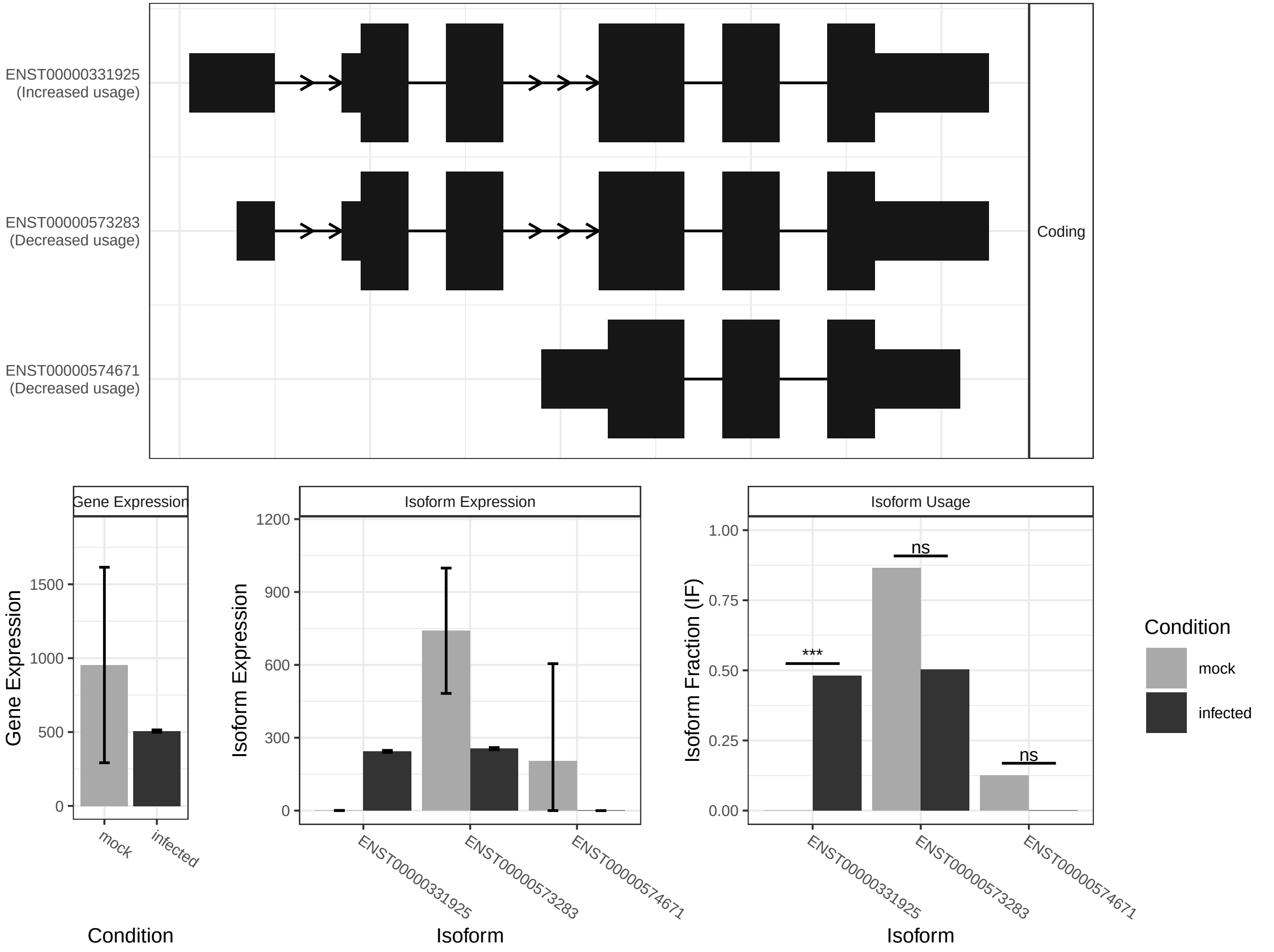

The isoform switch in ENSG00000260456 gene (mock vs infected)

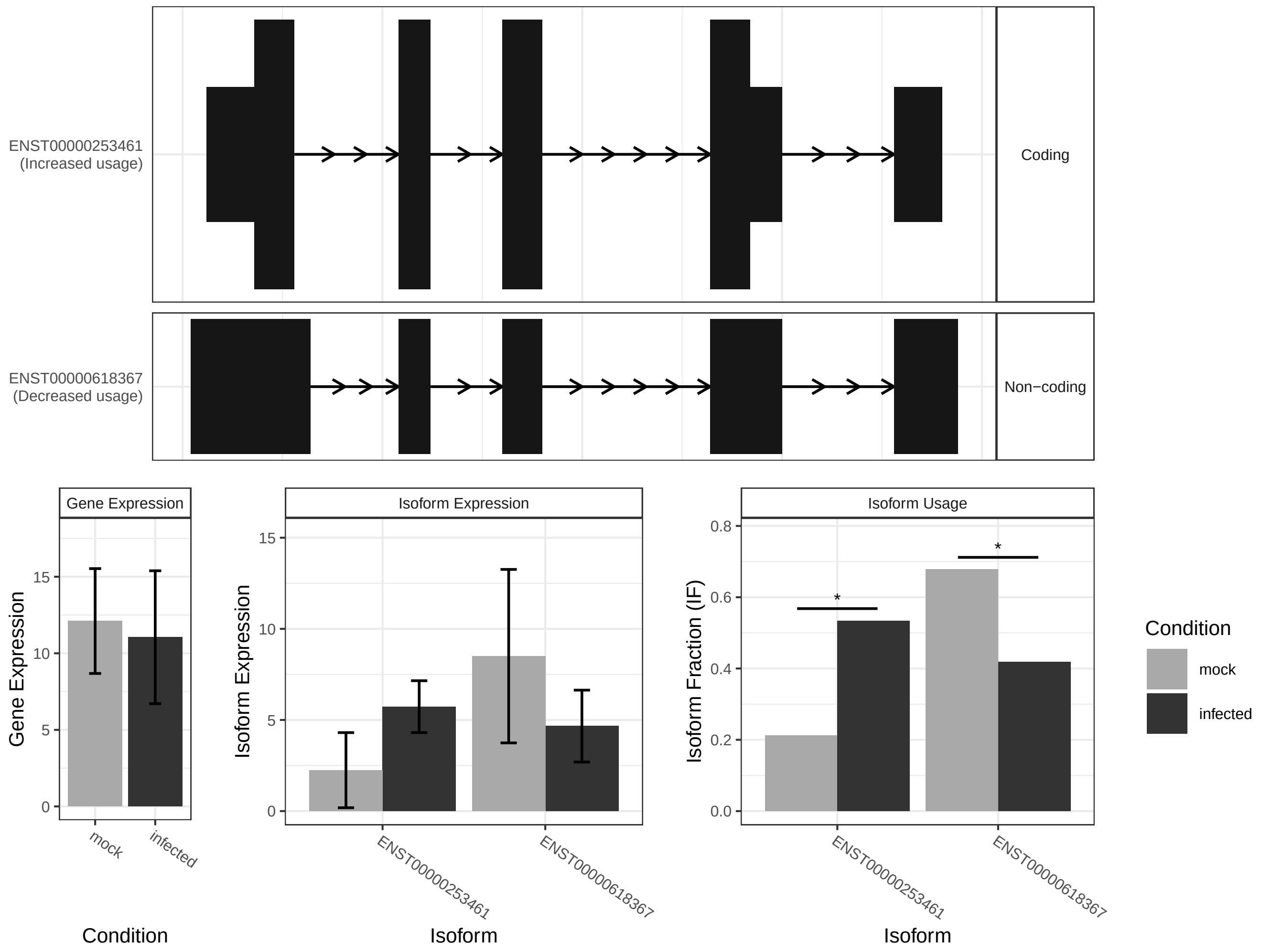

Supplementary Figure S7. Isoform switch analysis of the genes between mock and infected conditions, generated using the switchPlot function in IsoformSwitchAnalyzeR. The top panel provides a schematic representation of the gene's isoforms, with exons depicted as black boxes and introns as connecting lines. The lower left panel presents gene expression levels using a bar chart, where the bars represent the mean expression levels for the mock-treated and infected conditions. The lower middle panel shows isoform expression levels as a bar chart, representing the mean expression levels of each isoform across the two conditions. The lower right panel displays isoform usage fractions (IF) for the two isoforms, showing their relative contribution to the total gene expression. Statistical significance is indicated with an asterisk (*), while "ns" denotes a lack of significant difference.
